# Asymmetric one-pot sequential Friedel–Crafts-type alkylation and α-oxyamination catalyzed by a peptide and an enzyme

**DOI:** 10.3762/bjoc.8.152

**Published:** 2012-08-17

**Authors:** Kengo Akagawa, Ryota Umezawa, Kazuaki Kudo

**Affiliations:** 1 Institute of Industrial Science, University of Tokyo, 4-6-1 Komaba, Meguro-ku, Tokyo 153-8505, Japan

**Keywords:** Friedel–Crafts-type alkylation, laccase, one-pot reaction, organocatalysis, α-oxyamination, resin-supported peptide catalyst

## Abstract

In the presence of a peptide catalyst and the oxidative enzyme laccase, a one-pot sequential reaction including a Friedel–Crafts-type alkylation of α,β-unsaturated aldehydes followed by an α-oxyamination was realized. The reaction in aqueous solvent to promote the enzymatic oxidation, and the use of a peptide catalyst compatible with such conditions, were essential. The present sequential reaction afforded oxygen-functionalized indole or pyrrole derivatives in a highly enantioselective manner.

## Findings

Indole derivatives represent a class of biologically active compounds [1–3], and they often have chiral carbon chains attached to indole rings. A Friedel–Crafts-type asymmetric alkylation (FCAA) to indoles is a versatile method for synthesizing such chiral indole derivatives. To date, a number of FCAA reactions by either metal catalysts or organocatalysts have been reported [4–11]. Especially because organocatalysts have been demonstrated to possess a high feasibility for sequential reactions [12–16], it is expected that a sequential reaction including an organocatalytic FCAA step could provide highly functionalized indole compounds [17–20].

**Figure 1:**
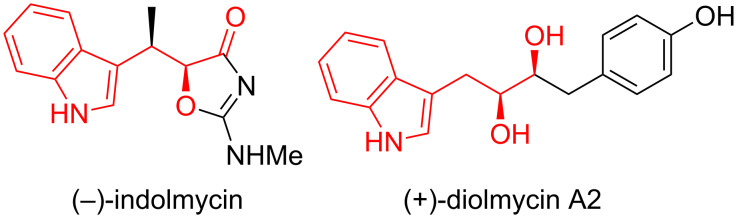
Oxygen-functionalized indole compounds.

Indoles with an oxygenated stereogenic carbon at the β-position of the ring, such as indolmycin [21–22] and diolmycin [23], are known as antibiotics (Figure 1). The framework of these compounds could be constructed though the conjugate addition of an indole to α,β-unsaturated aldehydes followed by oxygenation at the α-position of the carbonyl group. If such a sequence can be realized in a one-pot reaction, it would be a powerful method for the synthesis of oxy-functionalized indole derivatives with operational simplicity. To date, there has been no report on the FCAA reaction combined with an α-oxygenation of aldehydes [24–29] in a one-pot sequential reaction.

**Figure 2:**
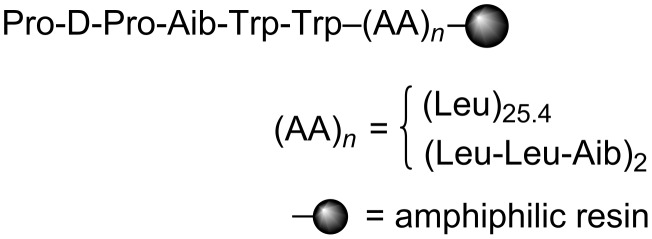
Resin-supported peptide catalyst.

Meanwhile, our group has developed resin-supported peptide catalysts (Figure 2) for several organic reactions in aqueous media [30–37]. Since these catalysts can be applicable for the FCAA reaction toward α,β-unsaturated aldehydes through iminium intermediates [32,37], and an asymmetric α-oxyamination of aldehydes via enamines [33], they are expected to be suitable for a one-pot sequential reaction to make the chiral indoles mentioned above. Herein, we report on an enantioselective synthesis of oxygenated indole compounds through a one-pot sequential FCAA/α-oxyamination catalyzed by the resin-supported peptide.

In a one-pot sequential reaction, the compatibility of reaction conditions in each step is important. Previously, we have reported a reaction system for the asymmetric α-oxyamination of aldehydes catalyzed by a peptide and an oxidizing enzyme, laccase [36,38]. Because the reaction conditions for that system are mild without employing a strong oxidant, we envisaged that the sequential FCAA/α-oxygenation could be attained by adopting the peptide-and-laccase-cocatalyzed oxyamination, even though indoles are generally prone to be oxidized [39–40]. By considering that enzymes promote reactions efficiently under aqueous conditions, we thought it necessary to conduct the one-pot reaction in aqueous media. Therefore, the reaction sequence shown in Table 1 was first examined in water. After the FCAA by peptide catalyst **1**, the α-oxyamination was successively performed by adding 2,2,6,6-tetramethylpiperidin-1-oxyl (TEMPO) and laccase directly to the reaction mixture. The desired two-step reaction product **4** was obtained with syn/anti ratio of 75:25, and the ee value of the syn diastereomer was 96% (Table 1, entry 1). It is noteworthy that the ee of the major diastereomer was higher than that of each single reaction [33,37]. Such enantio-enrichment is generally caused in consecutive catalytic asymmetric reactions through the formation of diastereomeric pairs [17,41]. From the viewpoint of reaction efficiency, the conversion in each step was low, presumably because of poor solubility of the substrates in water. Thus, use of the organic cosolvent THF was examined (Table 1, entries 2 to 5). As a result, the solvent system H_2_O/THF 2:1 was regarded as the optimum (Table 1, entry 4). When the reaction was performed under conditions with a higher content of THF, such as in the case of H_2_O/THF 1:1, the peptide/laccase-catalyzed oxidation did not proceed at all, due to inactivation of laccase (Table 1, entry 5). This indicates the importance of water as a solvent for realization of the present sequential reaction.

**Table 1:** One-pot sequential Friedel–Crafts-type alkylation/α-oxyamination.

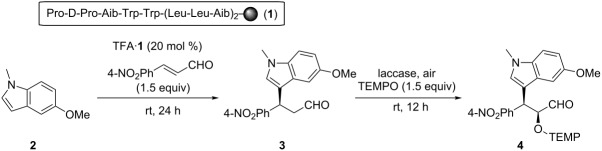				

entry	solvent	**2**:**3**:**4**^a^	syn/anti of **4**^a^	ee [%]^b^ of syn-isomer (anti-isomer)

1	H_2_O	67:14:19	75:25	96 (64)
2	H_2_O/THF 9:1	32:17:51	73:27	96 (62)
3	H_2_O/THF 5:1	44:10:46	76:24	97 (57)
4	H_2_O/THF 2:1	17:5:78	75:25	98 (56)
5	H_2_O/THF 1:1	15:85:0	–	–

^a^Determined by ^1^H NMR spectroscopy of crude mixture. ^b^Determined by HPLC analysis after being reduced to the corresponding alcohol.

**Scheme 1:**
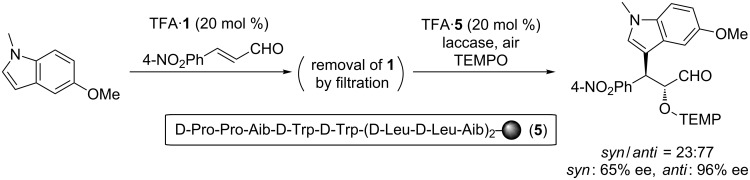
Effect of the stereostructure of the peptide catalyst.

To elucidate the origin of the stereocontrol in the present sequential reaction, the following control experiment was conducted. After the first FCAA reaction, peptide catalyst **1** was removed by filtration and another peptide catalyst **5**, which is the enantiomer of **1**, was added to promote the α-oxyamination (Scheme 1). In this case, the anti-isomer was obtained as a major diastereomer, and the ee value of the anti-product was high. The reversal of the diastereoselectivity along with the high ee of the major diastereomer demonstrates that the stereochemical course of the second-step α-oxyamination was determined mainly by the stereostructure of the peptide catalyst rather than by the chirality of the intermediate **3**.

Finally, other substrates were tested in the present one-pot sequential reaction system (Table 2). Several substituted indoles gave the products with high enantioselectivity (Table 2, entries 1 to 3). As an α,β-unsaturated aldehyde, 3-nitrocinnamaldehyde was also applicable (Table 2, entry 4). Other than indoles, a pyrrole compound could be employed as a starting nucleophile in the sequential FCAA/α-oxyamination (Table 2, entry 5).

**Table 2:** Examples of the one-pot synthesis of oxygenated heteroaromatic compounds.

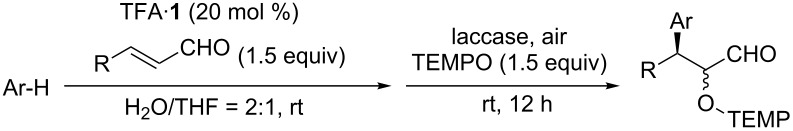					

entry	product	time (h) of first step	yield [%]^a^	syn/anti^b^	ee [%]^c^ of syn-isomer (anti-isomer)

1	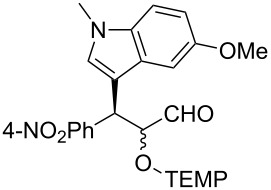 **4**	24	59	75:25	98 (56)
2	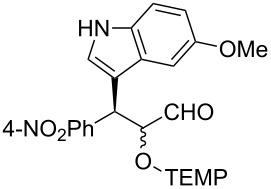 **6**	24	70	79:21	98 (73)
3	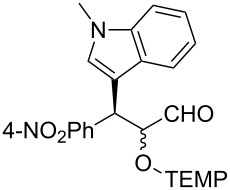 **7**	72	57	75:25	98 (55)
4	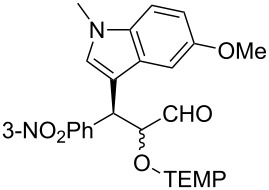 **8**	36	55	72:28	98 (61)
5	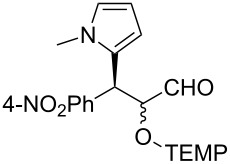 **9**	48	51	70:30	91 (30)

^a^Isolated yield. ^b^Determined by ^1^H NMR spectroscopy. ^c^Determined by HPLC analysis after being reduced to the corresponding alcohol.

In conclusion, the FCAA followed by the asymmetric α-oxyamination was realized in a one-pot reaction, by using a peptide catalyst and laccase. This sequential reaction afforded the oxygen-functionalized indole derivatives with high optical purity. By utilizing the wide applicability of peptide catalysts in aqueous media, and mild reaction conditions for enzymatic reactions, various types of new sequential reactions can be expected for producing highly functionalized compounds.

## Supporting Information

File 1 Typical experimental procedure, spectroscopic data for products, determination of stereochemistry, ^1^H and ^13^C NMR spectra and HPLC charts.
