# Supplementary material for: Asymmetric one-pot sequential Friedel–Crafts-type alkylation and α-oxyamination catalyzed by a peptide and an enzyme
Source: Beilstein J Org Chem. 2012 Aug 17;8:1333–7. doi: 10.3762/bjoc.8.152 (PMC3458757; doi:10.3762/bjoc.8.152)

Supporting Information

for

Asymmetric one-pot sequential Friedel–Crafts-type alkylation and α-oxyamination catalyzed by a peptide and an enzyme

Kengo Akagawa, Ryota Umezawa and Kazuaki Kudo*

Address: Institute of Industrial Science, University of Tokyo, 4-6-1 Komaba, Meguro-ku, Tokyo 153-8505, Japan

Email: Kazuaki Kudo* - kkudo@iis.u-tokyo.ac.jp

*Corresponding author

Typical experimental procedure, spectroscopic data for products, determination of stereochemistry, ^{1}H and ^{13}C NMR spectra and HPLC charts.

General information S2

Typical procedure and spectroscopic data for products S2

Determination of stereochemistry of products S8

^{1}H and ^{13}C NMR spectra S9

HPLC charts S19

General information

^{1}H and ^{13}C NMR spectra were recorded at 400 and 100 MHz respectively on a JEOL JNM-LA400 spectrometer, and chemical shifts were referenced to internal tetramethylsilane (TMS, δ = 0.0 ppm) for ^{1}H, and the central line of $CDCl_3$ (δ = 77.0 ppm) for ^{13}C. High-resolution FAB mass spectra were obtained on a JEOL JMS-600H mass spectrometer in positive-ionization mode by using 3-nitrobenzyl alcohol as a matrix. Polyethylene glycol 400 was added to the matrix as a mass calibrant. HPLC charts were recorded on a Shimadzu CLASS-VP system with a Chiralpak IA column (25 cm) and IA guard (1 cm), or Chiralpak AS-H column (25 cm) and AS-H guard (1 cm). The resin-supported peptide was synthesized according to our previous report [37]. Laccase was purchased from Sigma-Aldrich (product number: 53739, from Trametes versicolor, 93.6 U/mg) and used as received.

Typical procedure for the sequential FCAA/α-oxyamination (Table 2).

To a mixture of an indole compound (0.10 mmol), an aldehyde (0.15 mmol) and resin-supported TFA·Pro-D-Pro-Aib-$(Trp)_2$-$(Leu\text{-}Leu\text{-}Aib)_2$ (111 mg, 0.02 mmol of the terminal prolyl group) in THF (0.67 mL), was added water (1.34 mL), slowly. After the mixture had been stirred at room temperature for the given time, TEMPO (0.15 mmol) and laccase (0.5 mg) were added. Afterward the mixture was stirred for another 12 h, and then the peptide catalyst was filtered off and washed with diethyl ether. The filtrate solution was washed with water, and the organic layer was dried over anhydrous magnesium sulfate. After the removal of the solvent under reduced pressure, the crude product was purified by preparative TLC (hexane/ethyl acetate 2:1) to afford a diastereomeric mixture. Then, the product was dissolved in ethanol (ca. 2 mL), and sodium borohydride (0.3 mmol) was added. Afterward the mixture was stirred for 30 min, and then an aqueous saturated solution of ammonium chloride was added. The resulting solution was extracted with chloroform, and the organic layer was dried over anhydrous magnesium sulfate. After the removal of the solvent under reduced pressure, the crude product was purified by preparative TLC (hexane/ethyl acetate 1:1) to afford the corresponding alcohols of syn and anti-isomers separately (in Table 2, entry 4, two isomers could not be separated).

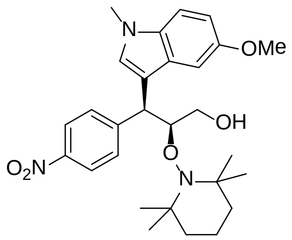

(2*S*,3*S*)-3-(5-Methoxy-1-methyl-1*H*-indol-3-yl)-3-(4-nitrophenyl)-2-(2,2,6,6-tetramethyl-piperidin-1-yloxy)propanol (*syn*-4, Table 2, entry 1).

^{1}H NMR ($CDCl_3$) δ 8.14–8.10 (m, 2H), 7.54–7.50 (m, 2H), 7.20–7.17 (m, 2H), 6.85 (dd, *J* = 8.7, 2.3 Hz, 1H), 6.62 (d, *J* = 2.8 Hz, 1H), 5.69 (br, 1H), 4.99 (ddd, *J* = 9.6, 3.7, 1.8 Hz, 1H), 4.49 (d, *J* = 3.7 Hz, 1H), 3.89 (dd, *J* = 12.4, 9.6 Hz, 1H), 3.78 (s, 3H), 3.78 (dd, *J* = 12.4, 1.8 Hz, 1H), 3.71 (s, 3H), 1.62–1.30 (m, 6H), 1.26 (s, 3H), 1.14 (s, 3H), 1.09 (s, 3H), 1.01 (s, 3H); ^{13}C NMR ($CDCl_3$) δ 153.79, 149.53, 146.55, 132.07, 129.80, 127.84, 127.82, 123.21, 111.84, 111.43, 110.07, 100.99, 82.72, 66.95, 62.03, 60.22, 55.83, 44.79, 40.53, 40.24, 34.66, 33.05, 32.12, 20.74, 20.31, 16.96; HRMS (FAB) *m*/*z*: calculated for $C_{28}H_{38}N_3O_5$ $[M + H]^+$: 496.2811, found 496.2820. Enantiomeric excess was determined by HPLC analysis (Chiralpak IA, hexane/2-propanol 90:10, 1.0 mL min^{-1}): t_R = 21.7 min (minor), 30.3 min (major).

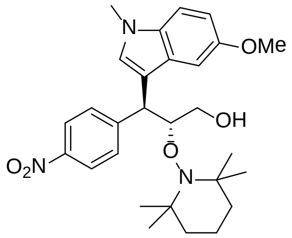

(2*R*,3*S*)-3-(5-Methoxy-1-methyl-1*H*-indol-3-yl)-3-(4-nitrophenyl)-2-(2,2,6,6-tetramethyl-piperidin-1-yloxy)propanol (*anti*-4, Table 2, entry 1).

^{1}H NMR ($CDCl_3$) δ 8.14–8.10 (m, 2H), 7.56–7.52 (m, 2H), 7.16 (d, *J* = 8.7 Hz, 1H), 7.10 (s, 1H), 6.87–6.82 (m, 2H), 6.12 (br, 1H), 5.00 (td, *J* = 9.2, 1.4 Hz, 1H), 4.17 (d, *J* = 9.2 Hz, 1H), 4.08 (dd, *J* = 12.4, 9.6 Hz, 1H), 3.91 (dd, *J* = 12.4, 1.4 Hz, 1H), 3.79 (s, 3H), 3.74 (s, 3H), 1.54–1.27 (m, 12H), 0.97 (s, 3H), 0.68 (s, 3H); ^{13}C NMR ($CDCl_3$) δ 153.94, 150.79, 146.23, 132.16, 129.26, 127.27, 126.58, 123.20, 113.01, 111.93, 110.18, 100.81, 82.00, 67.69, 61.95, 60.12, 55.92, 45.03, 40.33, 39.83, 34.61, 33.04, 32.42, 20.70, 20.18, 16.96; HRMS (FAB) *m*/*z*: calculated for $C_{28}H_{38}N_3O_5$ $[M + H]^+$: 496.2811, found 496.2811. Enantiomeric excess was determined by HPLC analysis (Chiralpak IA, hexane/2-propanol 90:10, 1.0 mL min^{-1}): t_R = 31.0 min (minor), 46.6 min (major).

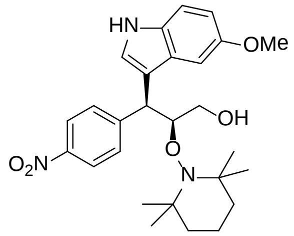

(2*S*,3*S*)-3-(5-Methoxy-1*H*-indol-3-yl)-3-(4-nitrophenyl)-2-(2,2,6,6-tetramethylpiperidin-1-yloxy)propanol (*syn*-6, Table 2, entry 2).

^{1}H NMR ($CDCl_3$) δ 8.16 (br, 1H), 8.15–8.10 (m, 2H), 7.55–7.50 (m, 2H), 7.36 (s, 1H), 7.25 (d, *J* = 8.7 Hz, 1H), 6.82 (dd, *J* = 8.7, 2.3 Hz, 1H), 6.65 (d, *J* = 2.3 Hz, 1H), 5.77 (br, 1H), 5.01 (ddd, *J* = 9.6, 4.1, 1.8 Hz, 1H), 4.49 (d, *J* = 4.1 Hz, 1H), 3.94 (dd, *J* = 12.4, 9.6 Hz, 1H), 3.77 (dd, *J* = 12.4, 1.8 Hz, 1H), 3.72 (s, 3H), 1.60–1.30 (m, 6H), 1.25 (s, 3H), 1.12 (s, 3H), 1.12 (s, 3H), 0.98 (s, 3H); ^{13}C NMR ($CDCl_3$) δ 154.02, 149.40, 146.57, 131.00, 129.76, 127.55, 123.25, 123.22, 113.31, 112.31, 111.88, 100.86, 82.78, 67.00, 62.07, 60.26, 55.78, 44.83, 40.52, 40.22, 34.60, 32.14, 20.74, 20.30, 16.93; HRMS (FAB) *m*/*z*: calculated for $C_{27}H_{36}N_3O_5$ $[M + H]^+$: 482.2655, found 482.2654. Enantiomeric excess was determined by HPLC analysis (Chiralpak AS-H, hexane/2-propanol 90:10, 1.0 mL min^{-1}): t_R = 31.5 min (minor), 37.9 min (major).

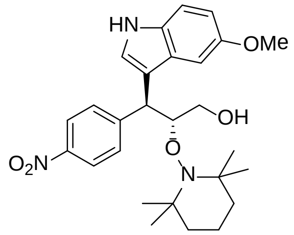

(2*R*,3*S*)-3-(5-Methoxy-1*H*-indol-3-yl)-3-(4-nitrophenyl)-2-(2,2,6,6-tetramethylpiperidin-1-yloxy)propanol (*anti*-4, Table 2, entry 2).

^{1}H NMR ($CDCl_3$) δ 8.18 (br, 1H), 8.14–8.10 (m, 2H), 7.57–7.53 (m, 2H), 7.27 (d, *J* = 1.8 Hz, 1H), 7.23 (d, *J* = 9.2 Hz, 1H), 6.85–6.81 (m, 2H), 6.21 (br, 1H), 5.04 (td, *J* = 9.2, 1.4 Hz, 1H), 4.20 (d, *J* = 9.2 Hz, 1H), 4.09 (dd, *J* = 11.9, 9.2 Hz, 1H), 3.92 (dd, *J* = 11.9, 1.4 Hz, 1H), 3.78 (s, 3H), 1.56–1.25 (m, 12H), 0.98 (s, 3H), 0.70 (s, 3H); ^{13}C NMR ($CDCl_3$) δ 154.14, 150.39, 146.32, 131.15, 129.40, 126.93, 123.18, 122.10, 114.89, 112.40, 112.00, 100.72, 81.89, 67.66, 62.01, 60.22, 55.85, 45.10, 40.34, 39.86, 34.55, 32.35, 20.72, 20.23, 16.93; HRMS (FAB) *m*/*z*: calculated for $C_{27}H_{36}N_3O_5$ $[M + H]^+$: 482.2655, found 482.2651. Enantiomeric excess was determined by HPLC analysis (Chiralpak IA, hexane/2-propanol 90:10, 1.0 mL min^{-1}): t_R = 36.0 min (minor), 42.4 min (major).

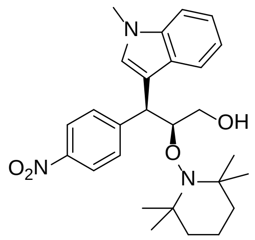

(2*S*,3*S*)-3-(1-Methyl-1*H*-indol-3-yl)-3-(4-nitrophenyl)-2-(2,2,6,6-tetramethylpiperidin-1-yloxy)propanol (*syn*-7, Table 2, entry 3).

^{1}H NMR ($CDCl_3$) δ 8.13–8.09 (m, 2H), 7.54–7.50 (m, 2H), 7.30 (d, *J* = 8.2 Hz, 1H), 7.24–7.17 (m, 3H), 7.00 (td, *J* = 7.6, 0.9 Hz, 1H), 5.69 (br, 1H), 5.04–4.98 (m, 1H), 4.56 (d, *J* = 3.6 Hz, 1H), 3.93–3.75 (m, 2H), 3.82 (s, 3H), 1.60–1.31 (m, 6H), 1.27 (s, 3H), 1.15 (s, 3H), 1.10 (s, 3H), 1.02 (s, 3H); ^{13}C NMR ($CDCl_3$) δ 149.54, 146.56, 136.65, 129.80, 127.50, 127.22, 123.20, 121.94, 119.14, 118.87, 112.03, 109.28, 82.76, 66.93, 62.08, 60.27, 44.78, 40.51, 40.23, 34.61, 32.86, 32.09, 20.73, 20.31, 16.94; HRMS (FAB) *m*/*z*: calculated for $C_{27}H_{36}N_3O_4$ $[M + H]^+$: 466.2706, found 466.2707. Enantiomeric excess was determined by HPLC analysis (Chiralpak IA, hexane/2-propanol 90:10, 1.0 mL min^{-1}): t_R = 10.9 min (minor), 15.7 min (major).

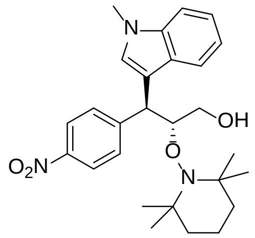

(2*R*,3*S*)-3-(1-Methyl-1*H*-indol-3-yl)-3-(4-nitrophenyl)-2-(2,2,6,6-tetramethylpiperidin-1-yloxy)propanol (*anti*-7, Table 2, entry 3).

^{1}H NMR ($CDCl_3$) δ 8.13–8.09 (m, 2H), 7.57–7.53 (m, 2H), 7.43 (d, *J* = 7.8 Hz, 1H), 7.27 (d, *J* = 8.2 Hz, 1H), 7.20 (t, *J* = 8.2 Hz, 1H), 7.14 (s, 1H), 7.05 (dd, *J* = 8.2, 7.8 Hz, 1H), 6.16 (br, 1H), 5.04 (td, *J* = 9.2, 1.4 Hz, 1H), 4.25 (d, *J* = 9.2 Hz, 1H), 4.08 (dd, *J* = 11.9, 9.2 Hz, 1H), 3.90 (dd, *J* = 11.9, 1.4 Hz, 1H), 3.78 (s, 3H), 1.55–1.28 (m, 12H), 0.98 (s, 3H), 0.69 (s, 3H); ^{13}C NMR ($CDCl_3$) δ 150.79, 146.23, 136.73, 129.27, 126.89, 126.01, 123.21, 122.14, 119.36, 118.59, 113.57, 109.43, 82.06, 67.64, 62.06, 60.26, 45.04, 40.29, 39.80, 34.55, 32.86, 32.36, 20.69, 20.18, 16.93; HRMS (FAB) *m*/*z*: calculated for $C_{27}H_{36}N_3O_4$ $[M + H]^+$: 466.2706, found 466.2699. Enantiomeric excess was determined by HPLC analysis (Chiralpak IA, hexane/2-propanol 90:10, 1.0 mL min^{-1}): t_R = 11.9 min (major), 13.6 min (minor).

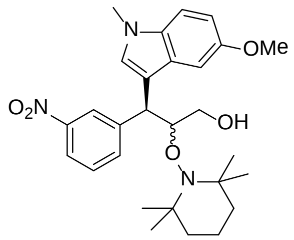

3-(5-Methoxy-1-methyl-1*H*-indol-3-yl)-3-(3-nitrophenyl)-2-(2,2,6,6-tetramethylpiperidin-1-yloxy)propanol [diastereomeric mixture (ca. 7:3)] (*syn*- and *anti*-8, Table 2, entry 4).

^{1}H NMR ($CDCl_3$) δ 8.32 (t, *J* = 2.1 Hz, 0.7 H), 8.29 (t, *J* = 2.1 Hz, 0.3 H), 8.06 (ddd, *J* = 8.2, 2.1, 0.9 Hz, 0.7 H), 8.02 (ddd, *J* = 8.2, 2.1, 0.9 Hz, 0.3 H), 7.70 (dm, *J* = 8.2 Hz, 0.3H), 7.64 (dm, *J* = 8.2 Hz, 0.7H), 7.41 (t, *J* = 8.2 Hz, 1H), 7.21–7.13 (m. 2H), 6.87–6.83 (m, 1.3H), 6.65 (d, *J* = 2.7 Hz, 0.7H), 6.11 (br, 0.3H), 5.70 (br, 0.7H), 5.02–4.95 (m, 1H), 4.49 (d, *J* = 3.7 Hz, 0.7H), 4.18 (d, *J* = 8.7 Hz, 0.3H), 4.06 (dd, *J* = 12.4, 9.6 Hz, 0.3H), 3.93–3.85 (m, 1H), 3.80–3.70 (m, 6.7H), 1.53–1.25 (m, 9.9H), 1.15 (s, 2.1H), 1.06 (s, 2.1H), 1.04 (s, 2.1H), 0.97 (s, 0.9H), 0.69 (s, 0.9H); ^{13}C NMR ($CDCl_3$) δ 153.93, 153.80, 147.99, 147.93, 144.88, 144.00, 135.07, 134.76, 132.18, 132.08, 128.83, 128.68, 127.92, 127.86, 127.23, 126.69, 123.94, 123.55, 121.62, 121.26, 113.21, 111.99, 111.86, 111.47, 110.19, 110.09, 100.97, 100.73, 82.62, 81.82, 67.73, 67.00, 62.03, 61.90, 60.21, 60.13, 55.94, 55.86, 44.78, 44.54, 40.58, 40.38, 40.29, 39.89, 34.64, 34.60, 33.06, 32.33, 32.07, 20.68, 20.60, 20.27, 20.08, 16.97; HRMS (FAB) *m*/*z*: calculated for $C_{28}H_{38}N_3O_5$ $[M + H]^+$: 496.2811, found 496.2814. Enantiomeric excess was determined by HPLC analysis (Chiralpak IA, hexane/2-propanol 90:10, 0.3 mL min^{-1}): t_R = 58.7 min (syn, minor), 60.9 min (syn, major), 80.3 min (anti, minor), 90.7 min (anti, major).

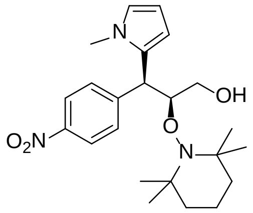

(2*S*,3*S*)-3-(1-Methyl-1*H*-pyrrol-2-yl)-3-(4-nitrophenyl)-2-(2,2,6,6-tetramethylpiperidin-1-yloxy)propanol (*syn*-9, Table 2, entry 5).

^{1}H NMR ($CDCl_3$) δ 8.15–8.11 (m, 2H), 7.38–7.34 (m, 2H), 6.54 (dd, *J* = 2.8, 1.8 Hz, 1H), 6.36 (dd, *J* = 3.6, 1.8 Hz, 1H), 6.13 (dd, *J* = 3.6, 2.8 Hz, 1H), 5.63 (br, 1H), 4.91 (ddd, *J* = 9.6, 4.6, 1.8 Hz, 1H), 4.21 (d, *J* = 4.6 Hz, 1H), 3.79 (dd, *J* = 12.4, 9.6 Hz, 1H), 3.69 (dd, *J* = 12.4, 1.8 Hz, 1H), 3.29 (s, 3H), 1.60–1.23 (m, 9H), 1.17 (s, 3H), 1.15 (s, 3H), 0.97 (s, 3H); ^{13}C NMR ($CDCl_3$) δ 147.63, 146.82, 129.84, 129.73, 123.50, 122.23, 107.98, 106.87, 82.53, 66.70, 62.07, 60.23, 45.39, 40.56, 40.25, 34.60, 34.02, 32.22, 20.64, 20.18, 16.96; HRMS (FAB) *m*/*z*: calculated for $C_{23}H_{34}N_3O_4$ [M + H]$^+$: 416.2549, found 416.2543. Enantiomeric excess was determined by HPLC analysis (Chiralpak IA, hexane/2-propanol 90:10, 1.0 mL min^{-1}): t_R = 11.4 min (major), 13.2 min (minor).

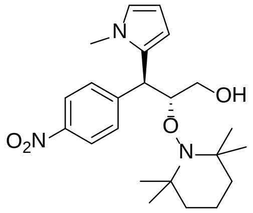

(2*R*,3*S*)-3-(1-Methyl-1*H*-pyrrol-2-yl)-3-(4-nitrophenyl)-2-(2,2,6,6-tetramethylpiperidin-1-yloxy)propanol (*anti*-9, Table 2, entry 5).

^{1}H NMR ($CDCl_3$) δ = 8.15–8.11 (m, 2H), 7.41–7.37 (m, 2H), 6.50 (dd, *J* = 2.7, 1.6 Hz, 1H), 6.31 (dd, *J* = 3.7, 1.6 Hz, 1H), 6.11 (dd, *J* = 3.7, 2.7 Hz, 1H), 6.00 (br, 1H), 4.88 (td, *J* = 8.7, 2.3 Hz, 1H), 4.02 (dd, *J* = 11.9, 8.7 Hz, 1H), 3.98 (dd, *J* = 11.9, 2.3 Hz, 1H), 3.92 (d, *J* = 8.7 Hz, 1H), 3.31 (s, 3H), 1.52-1.20 (m, 12H), 0.94 (s, 3H), 0.60 (s, 3H); ^{13}C NMR ($CDCl_3$) δ = 148.53, 146.49, 130.51, 129.73, 123.24, 122.27, 107.12, 107.02, 81.55, 67.50, 61.94, 60.11, 45.37, 40.30, 39.85, 34.48, 33.85, 32.25, 20.80, 20.13, 16.93; HRMS (FAB) *m*/*z*: calculated for $C_{23}H_{34}N_3O_4$ [M + H]$^+$: 416.2549, found 416.2543. Enantiomeric excess was determined by HPLC analysis (Chiralpak IA, hexane/2-propanol 90:10, 1.0 mL min^{-1}): t_R = 9.0 min (minor), 9.9 min (major).

Determination of stereochemistry of products.

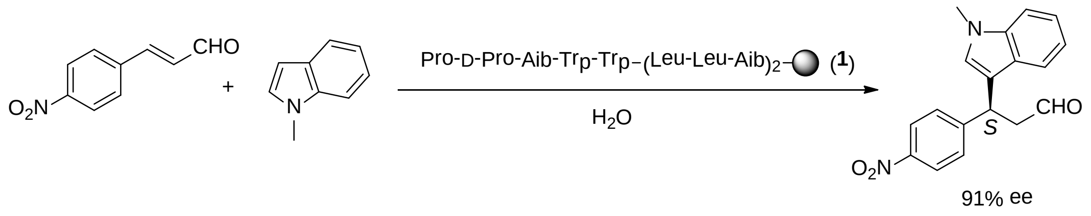

In the Friedel–Crafts-type alkylation with peptide catalyst **1** using the substrates shown in the above scheme, the absolute configuration of the major product can be assigned as the *S* form (*Tetrahedron Lett.* **2009,** *50,* 5602–5604). The diastereomers (ca. 75:25) obtained after the next α-oxyamination were reduced to the corresponding alcohols and separated from each other. The analyses by ^{1}H NMR and NOESY experiments were conducted to determine the relative configuration of the introduced oxyamino group.

With one isomer, a large ^{3}J coupling constant (9.2 Hz) between the protons shown below (with another isomer, ^{3}J = 3.6 Hz) and the H,H-NOEs between the 4-nitrophenyl group and the tetramethylpiperidinyl moiety were observed. This indicates that this compound is the anti isomer, and the other one is the syn isomer.

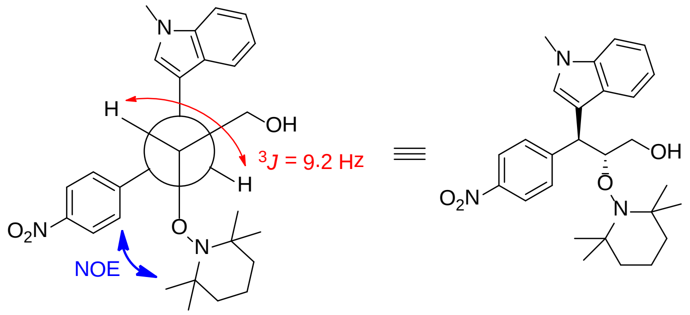

major conformation of anti isomer

Based on the mechanistic similarity of the reaction and in analogy with the NMR spectra of other products, the major configurations of all products were assigned as (2*S*,3*S*) or (2*R*,3*S*).

NOESY spectrum of anti-isomer.

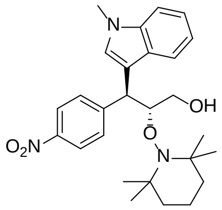

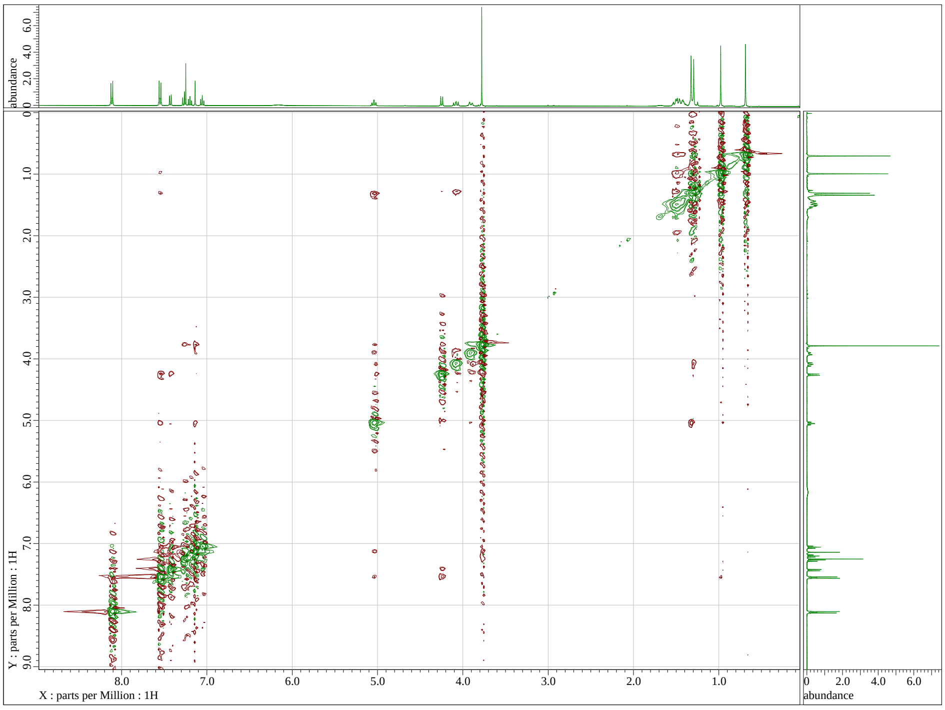

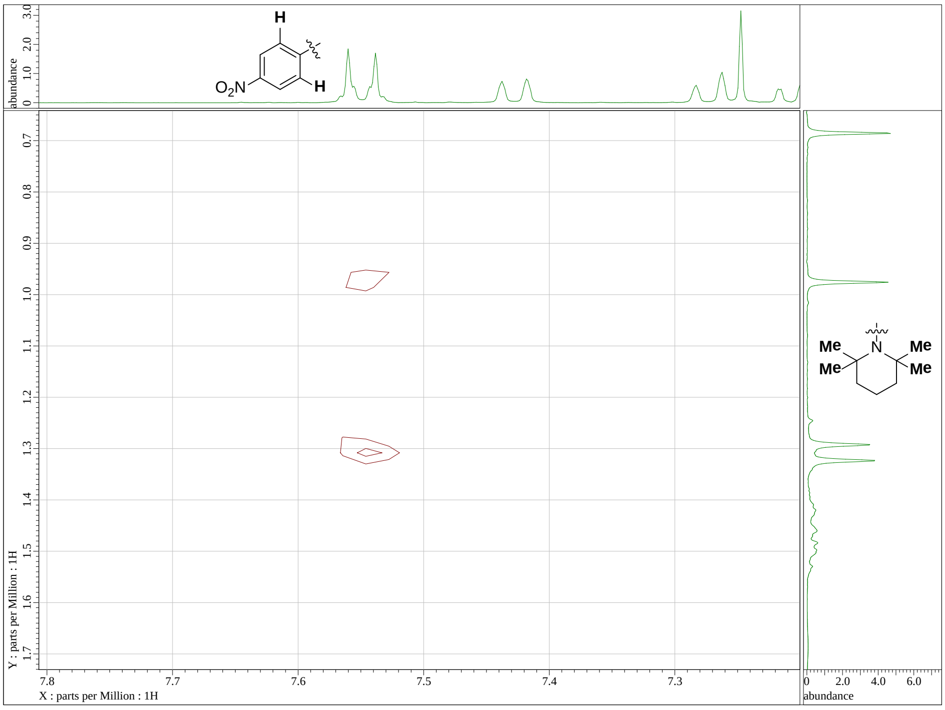

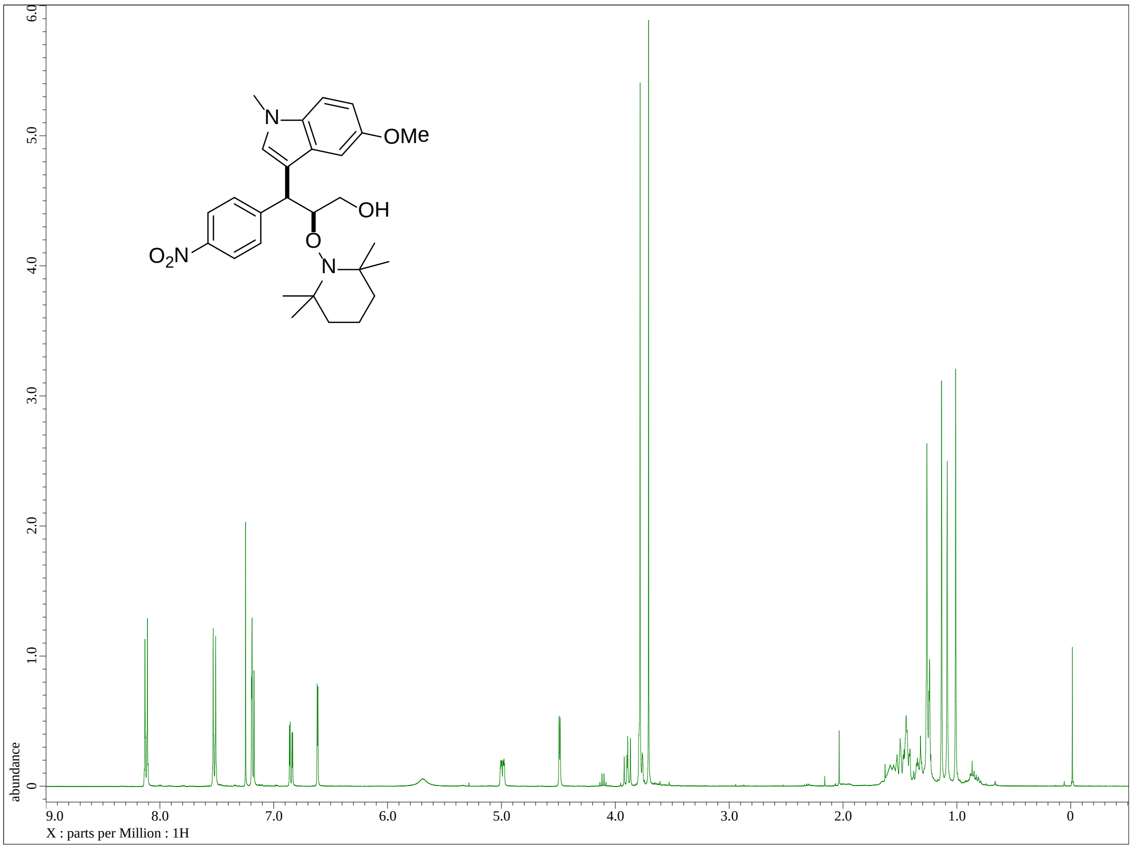
OMe
OH
O_2N
N
O
abundance
X : parts per Million : 1H

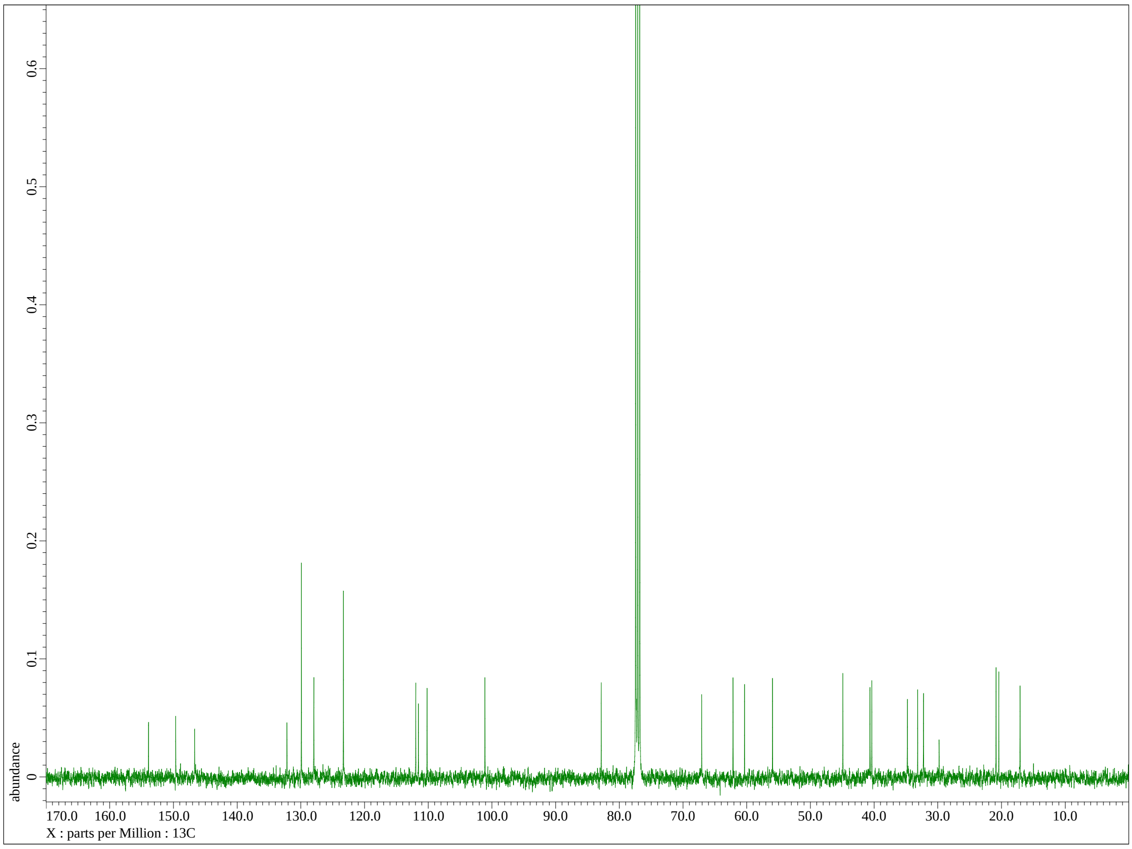
abundance
X : parts per Million : 13C

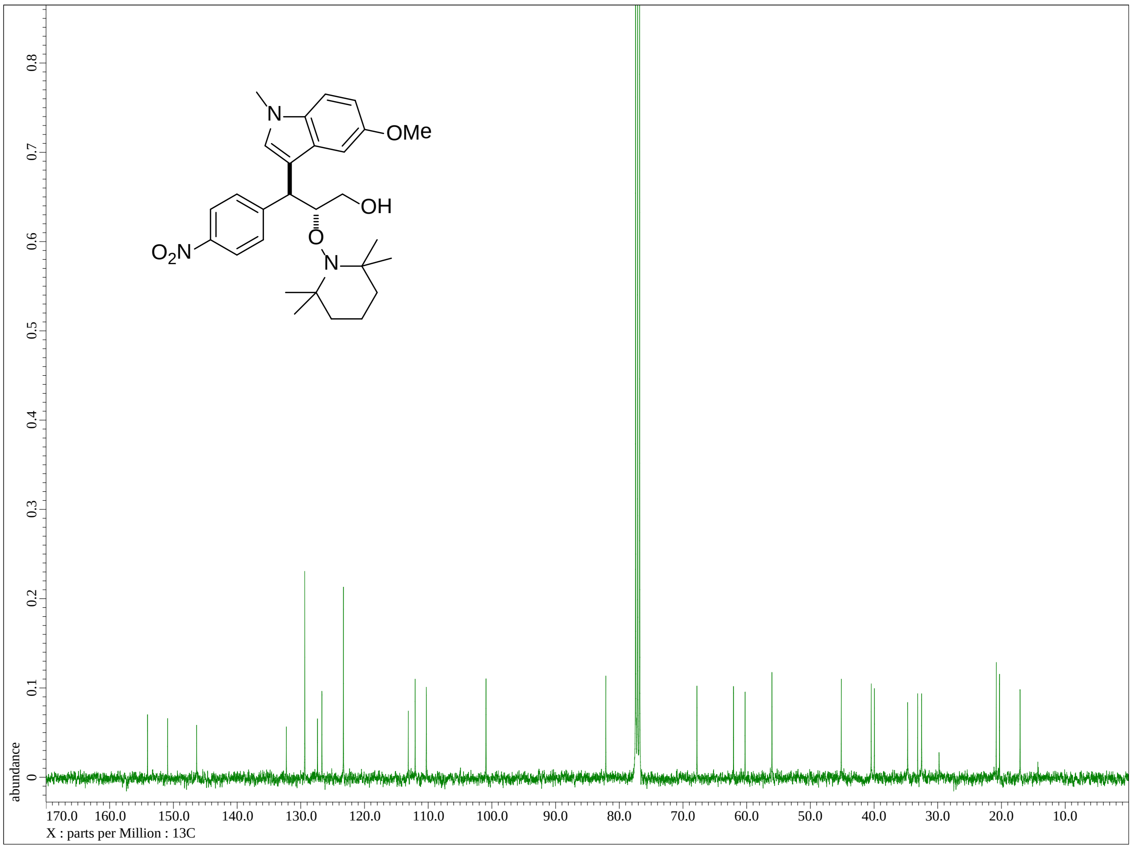

OMe
OH
O₂N
abundance
X : parts per Million : 13C

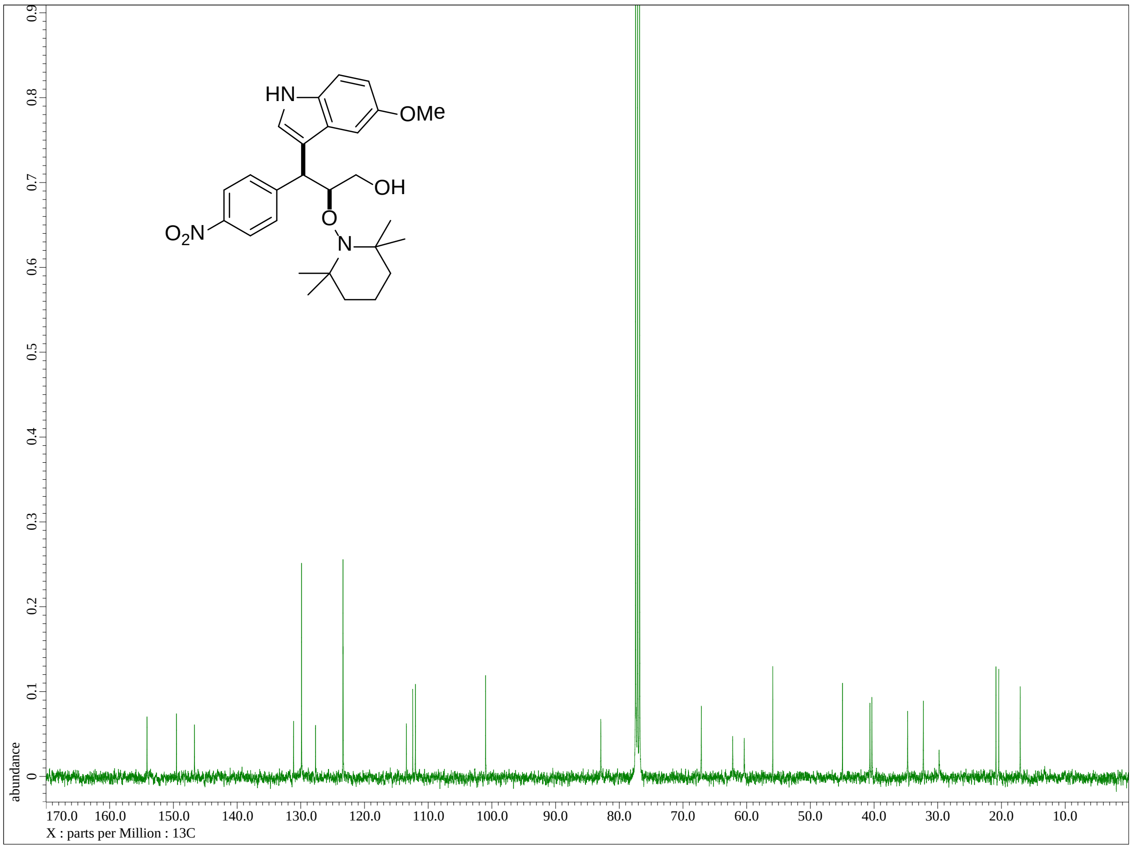
HN
OMe
OH
O_2N
O
N
abundance
X : parts per Million : 13C
170.0 160.0 150.0 140.0 130.0 120.0 110.0 100.0 90.0 80.0 70.0 60.0 50.0 40.0 30.0 20.0 10.0
0 0.1 0.2 0.3 0.4 0.5 0.6 0.7 0.8 0.9

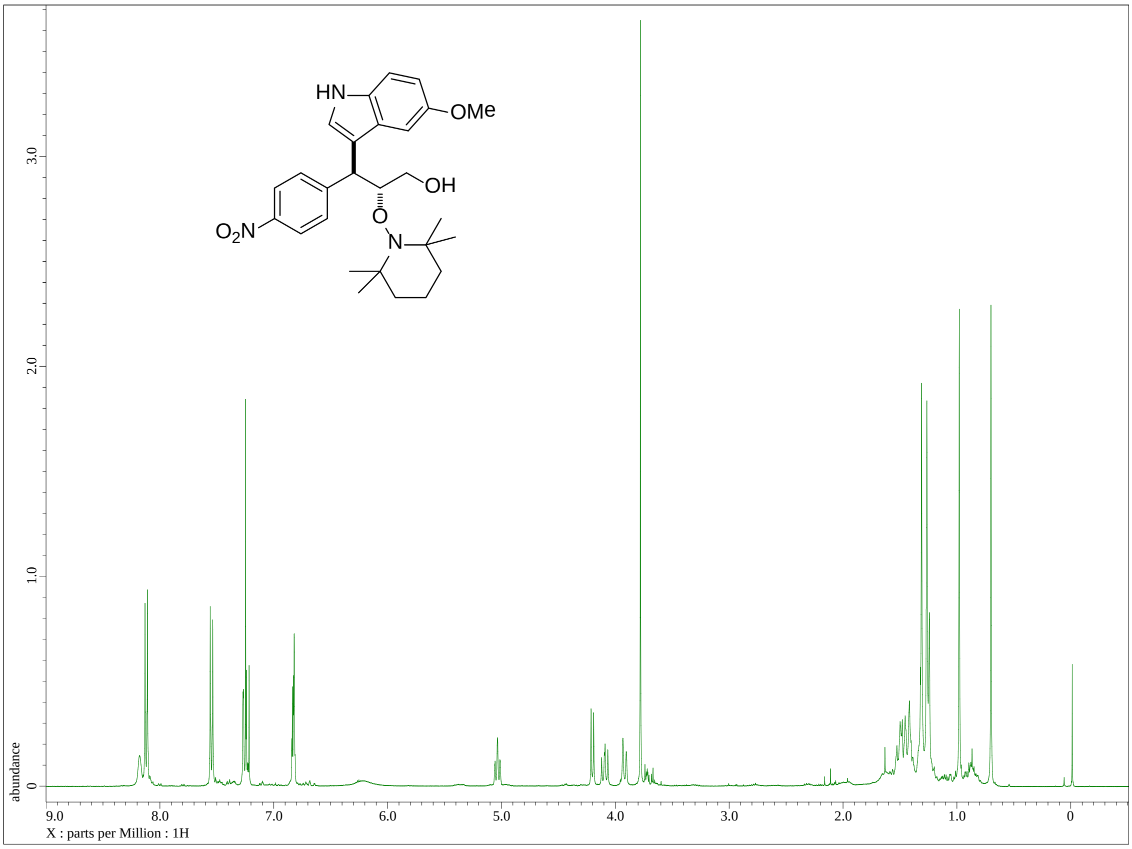
HN
OMe
OH
O_2N
O
N
abundance
3.0
2.0
1.0
0
9.0
8.0
7.0
6.0
5.0
4.0
3.0
2.0
1.0
0
X : parts per Million : 1H

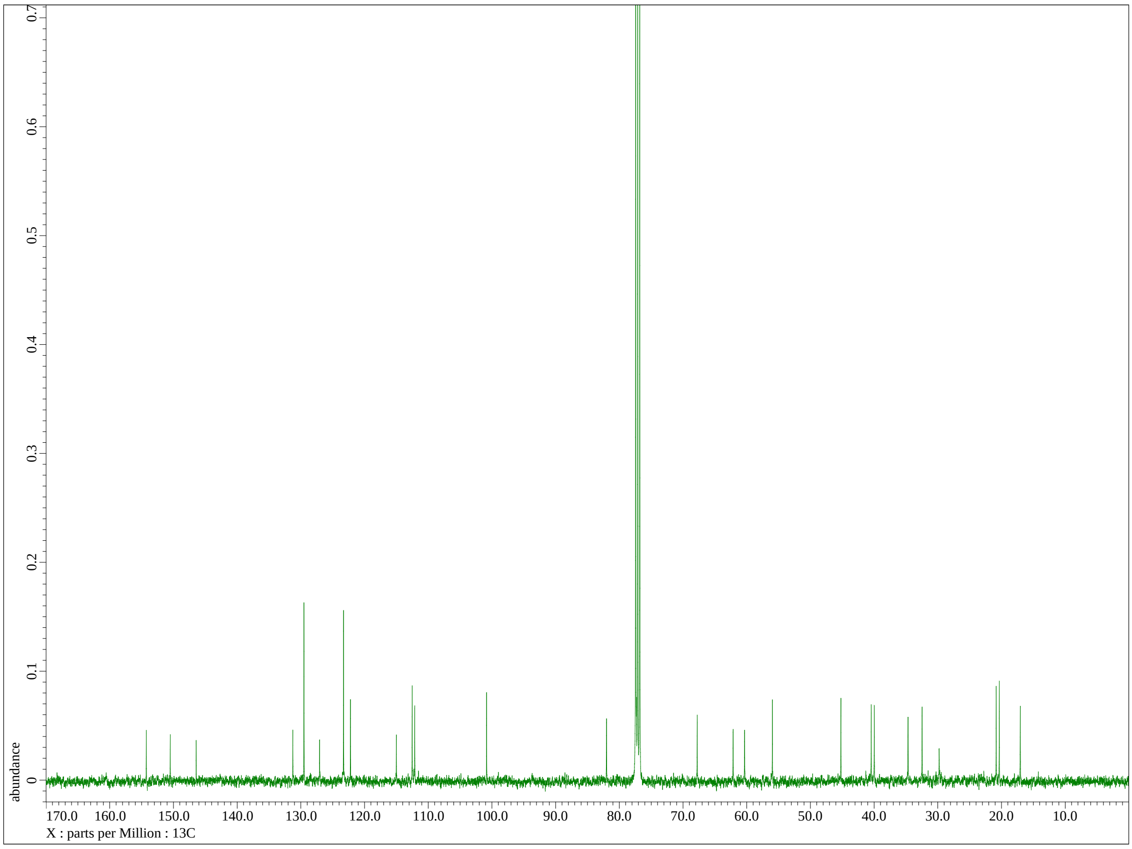
abundance
0.7
0.6
0.5
0.4
0.3
0.2
0.1
0
170.0
160.0
150.0
140.0
130.0
120.0
110.0
100.0
90.0
80.0
70.0
60.0
50.0
40.0
30.0
20.0
10.0
X : parts per Million : 13C

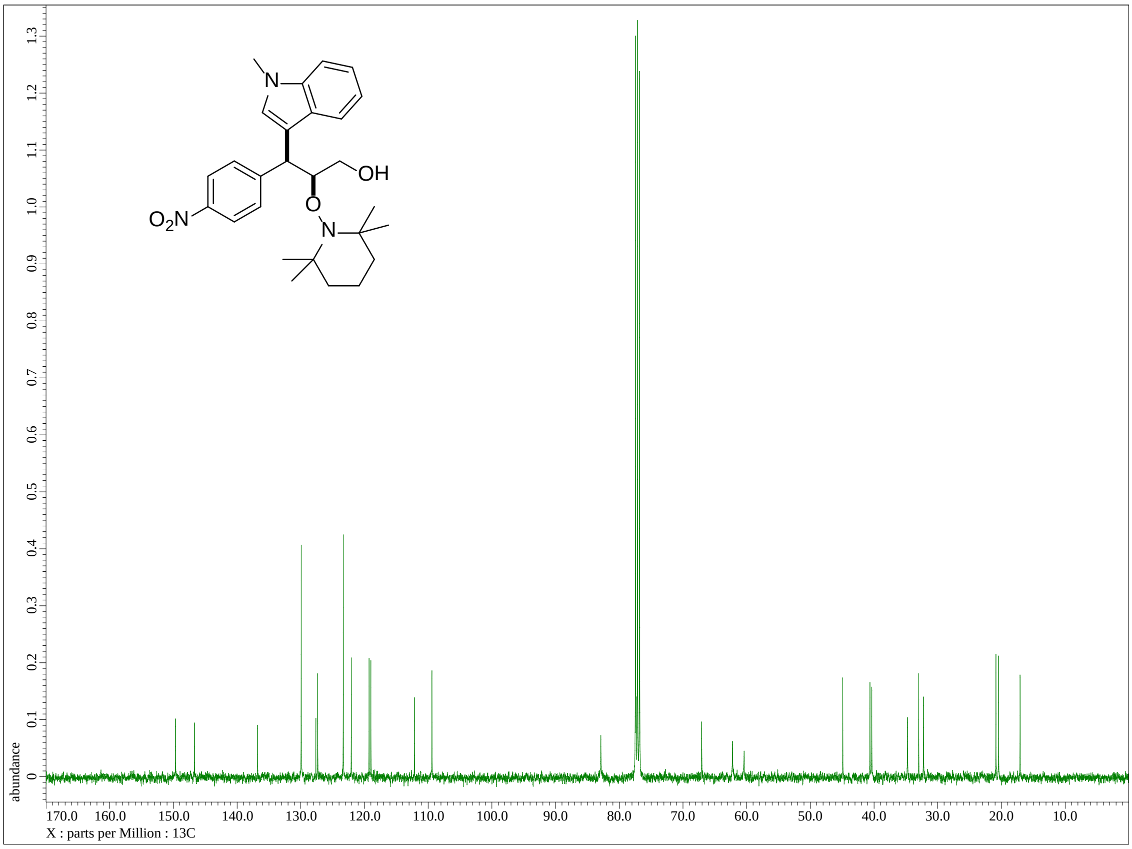

N
OH
O
N
O_2N
abundance
X : parts per Million : 13C

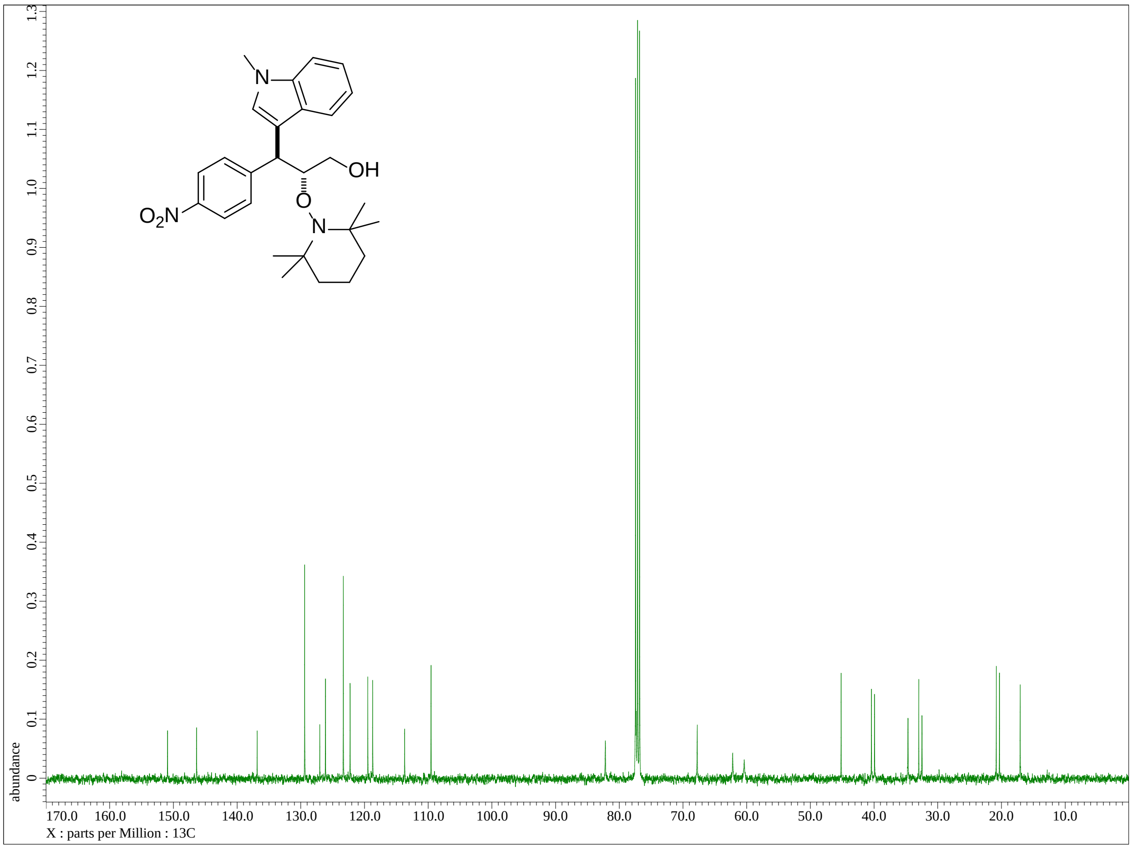

O2N
N
OH
O
N
abundance
X : parts per Million : 13C

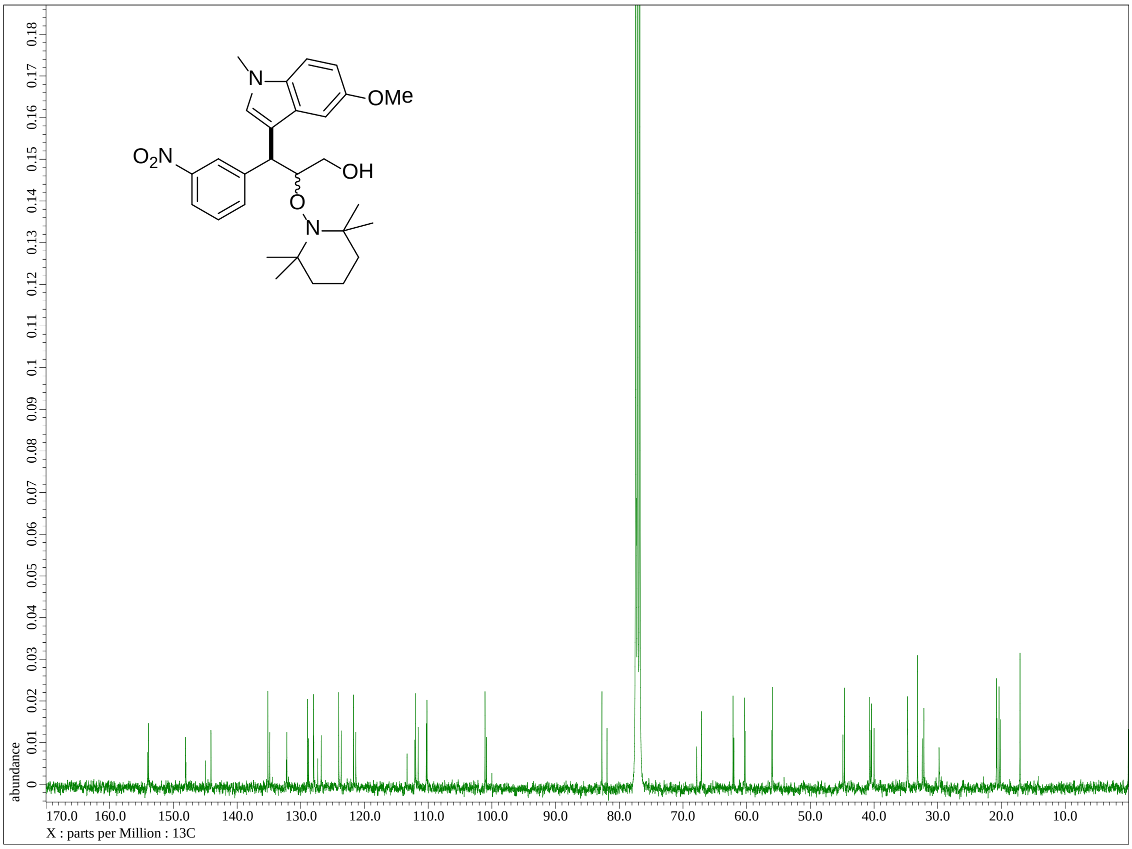

OMe
O2N
OH
N
O
N
abundance
0.18
0.17
0.16
0.15
0.14
0.13
0.12
0.11
0.1
0.09
0.08
0.07
0.06
0.05
0.04
0.03
0.02
0.01
0
170.0 160.0 150.0 140.0 130.0 120.0 110.0 100.0 90.0 80.0 70.0 60.0 50.0 40.0 30.0 20.0 10.0
X : parts per Million : 13C

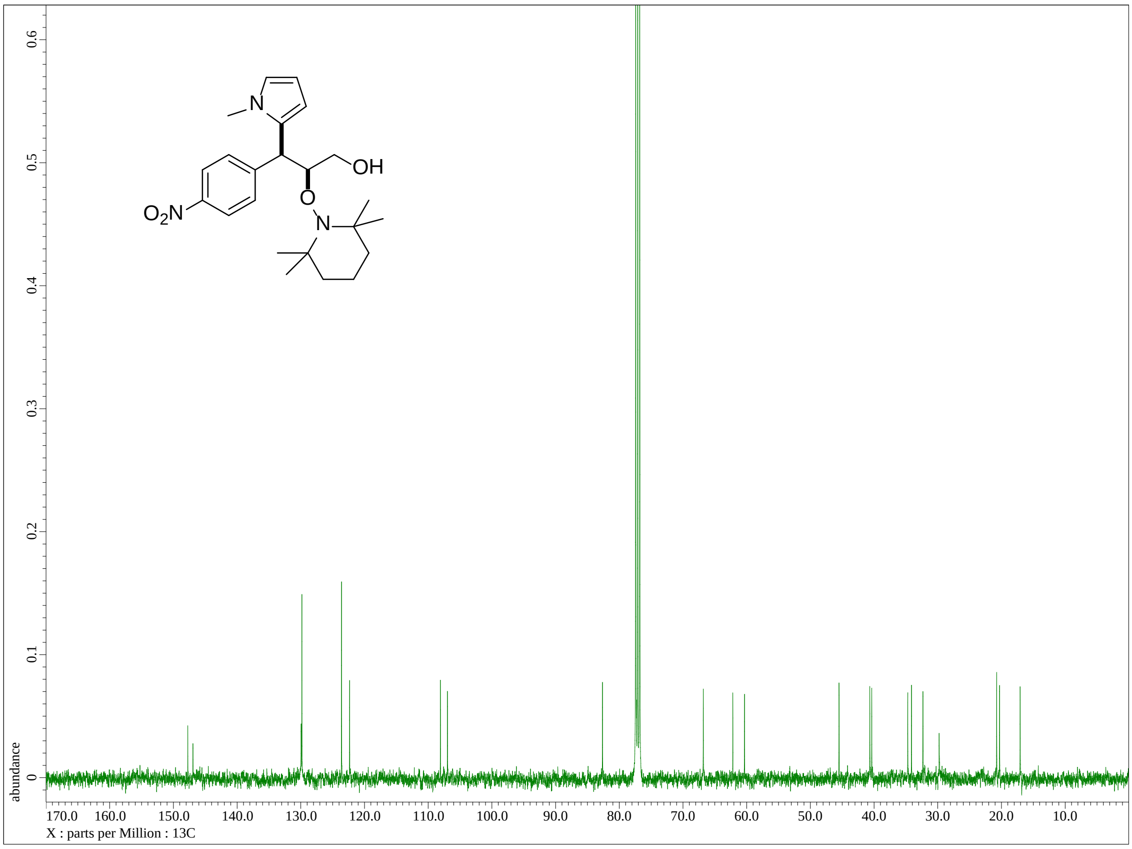
O2N
N
OH
O
N
abundance
0.6
0.5
0.4
0.3
0.2
0.1
0
170.0 160.0 150.0 140.0 130.0 120.0 110.0 100.0 90.0 80.0 70.0 60.0 50.0 40.0 30.0 20.0 10.0
X : parts per Million : 13C

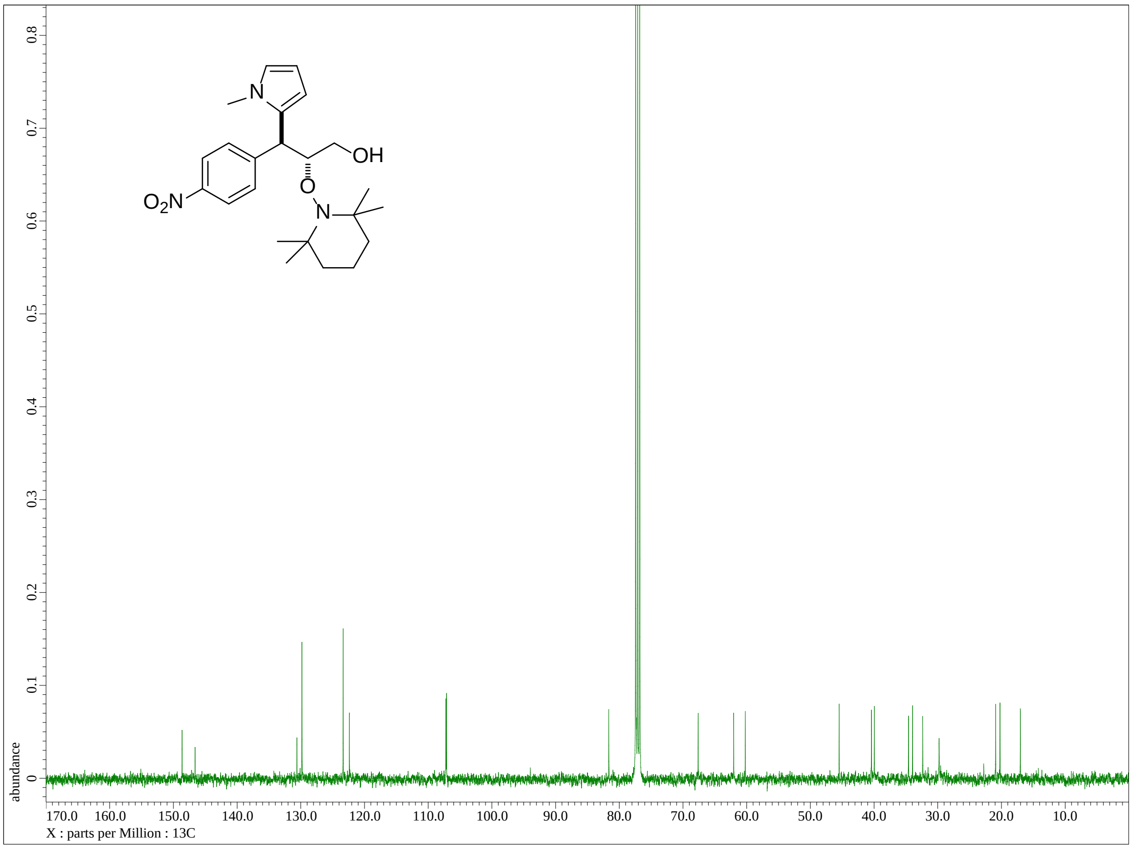

abundance
0.8
0.7
0.6
0.5
0.4
0.3
0.2
0.1
0
170.0
160.0
150.0
140.0
130.0
120.0
110.0
100.0
90.0
80.0
70.0
60.0
50.0
40.0
30.0
20.0
10.0
X : parts per Million : 13C
N
OH
O
N
O_2N

Chiralpak IA column, hexane/2-propanol 90:10, 1.0 mL min^{-1}

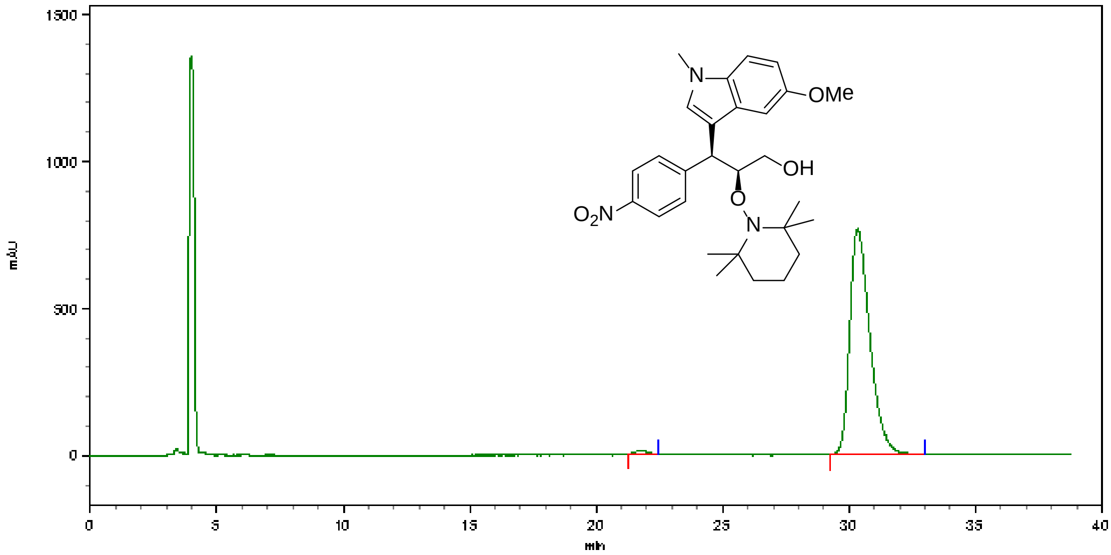

1: 220 nm, 8 nm

retention time	area	area%
21.749	482659	1.12
30.338	42698576	98.88
Total	43181235	100.00

racemic sample

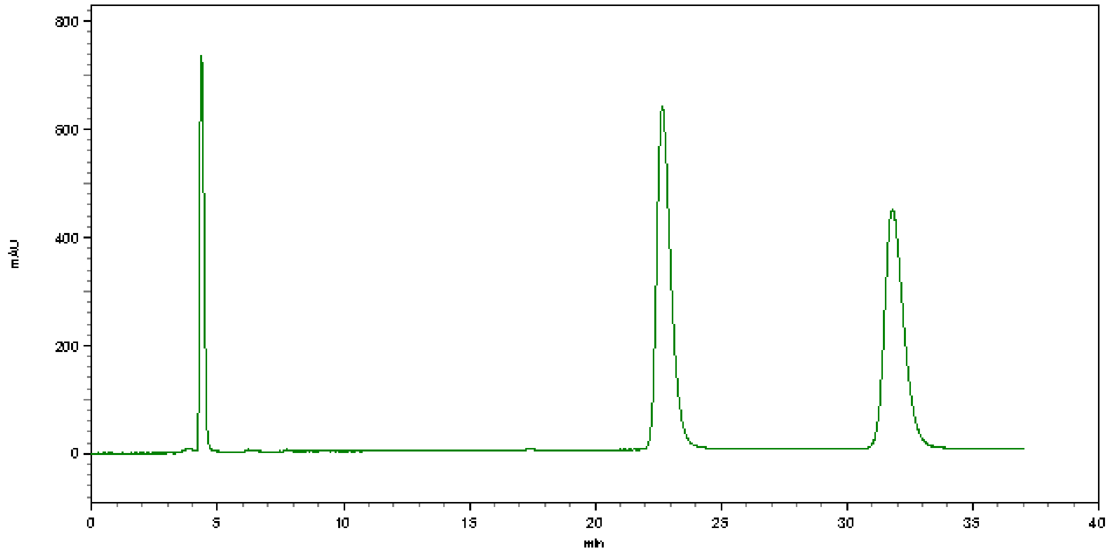

Chiralpak IA column, hexane/2-propanol 90:10, 1.0 mL min^{-1}

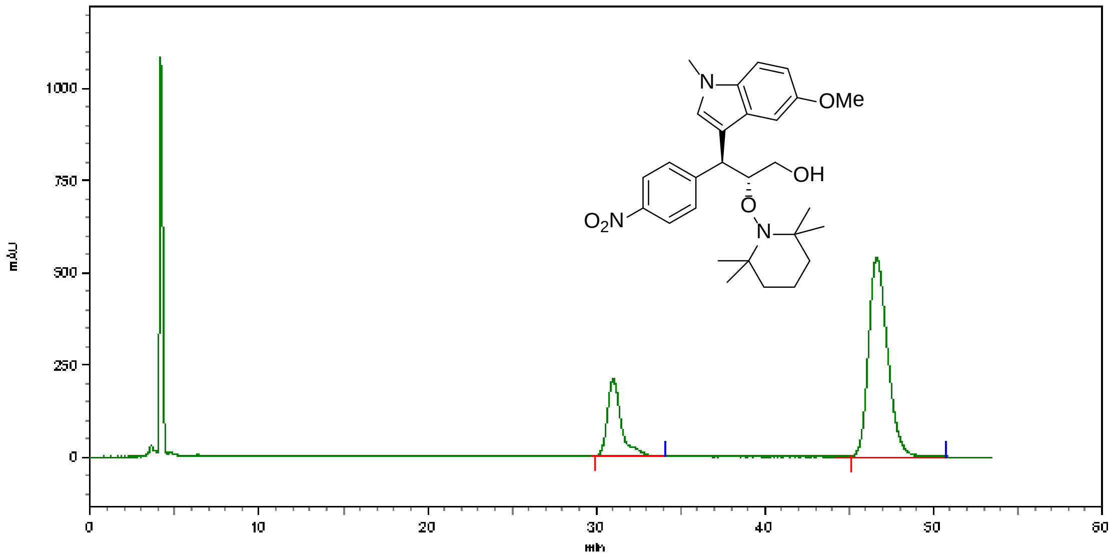

1: 220 nm, 8 nm

retention time	area	area%
31.010	11941288	22.03
46.623	42253256	77.97
Total	54194544	100.00

racemic sample

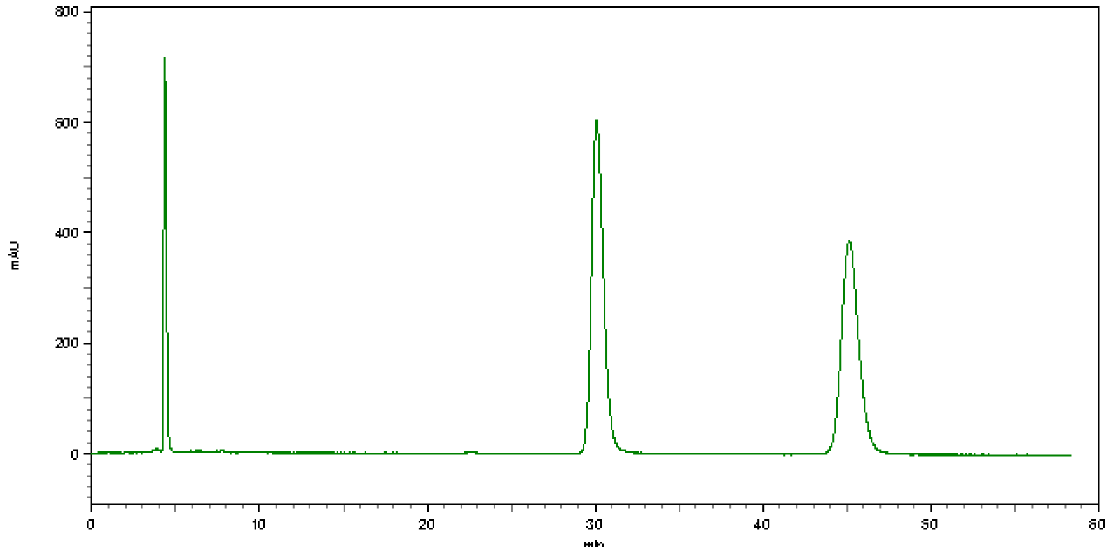

Chiralpak AS-H column, hexane/2-propanol 90:10, 1.0 mL min^{-1}

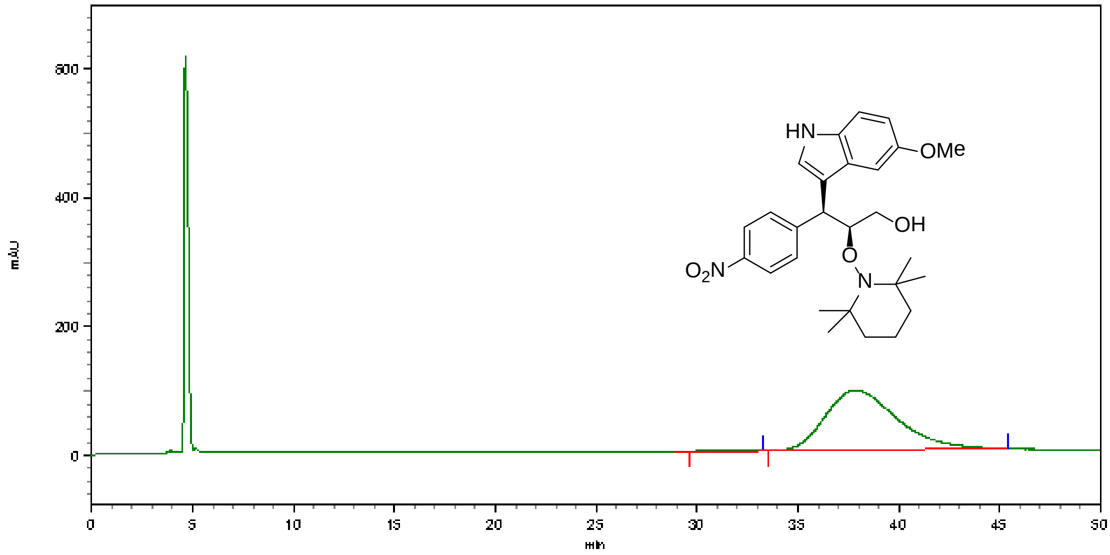

1: 220 nm, 8 nm

retention time	area	area%
31.529	227300	0.98
37.902	23042097	99.02
Total	23269397	100.00

racemic sample

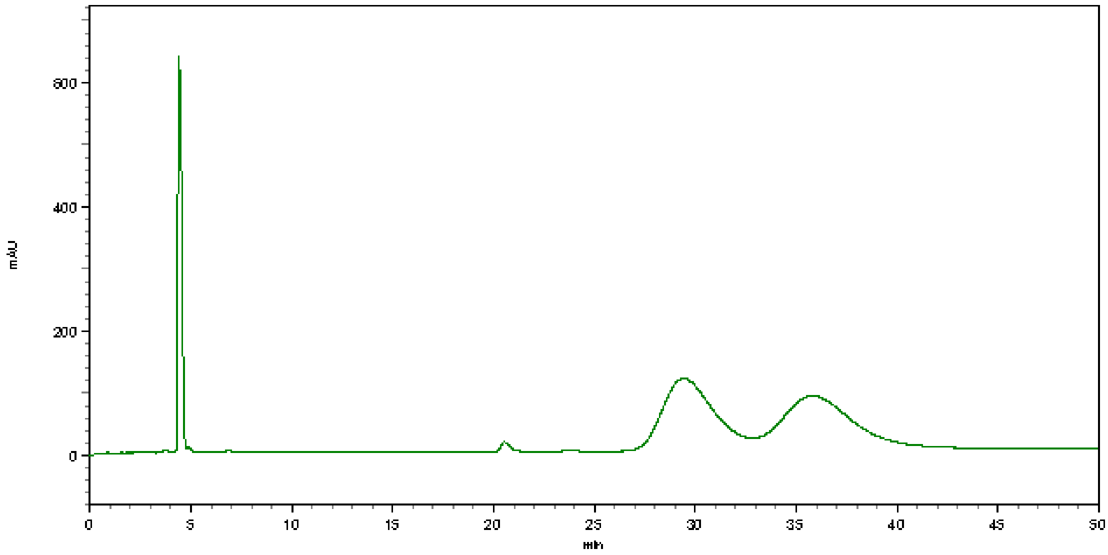

Chiralpak IA column, hexane/2-propanol 90:10, 1.0 mL min^{-1}

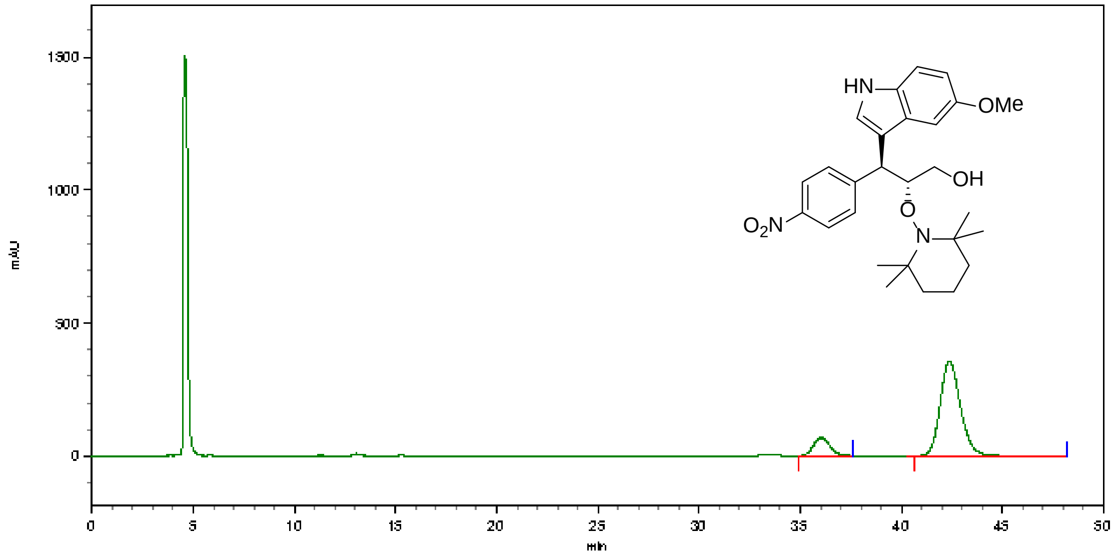

1: 220 nm, 8 nm

retention time	area	area%
36.042	3895937	13.42
42.368	25126043	86.58
Total	29021980	100.00

racemic sample

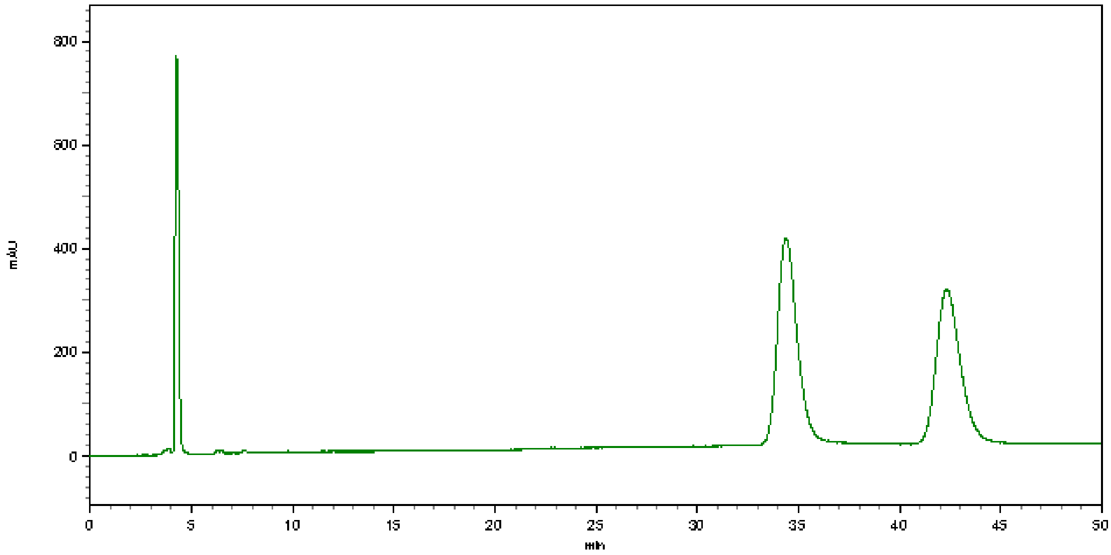

Chiralpak IA column, hexane/2-propanol 90:10, 1.0 mL min^{-1}

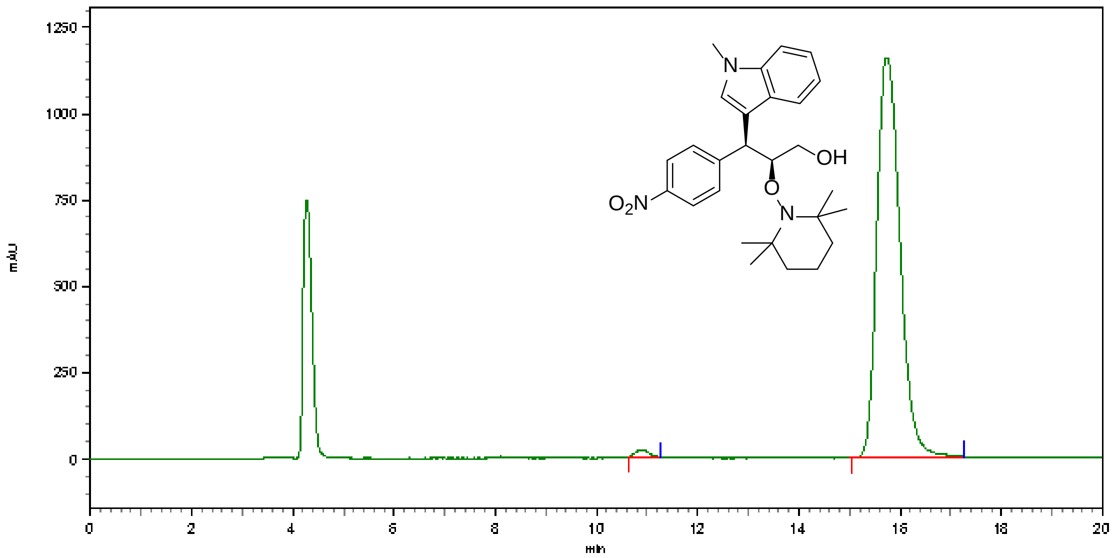

1: 220 nm, 8 nm

retention time	area	area%
10.884	397828	1.08
15.727	36566901	98.92

Total	36964729	100.00

racemic sample

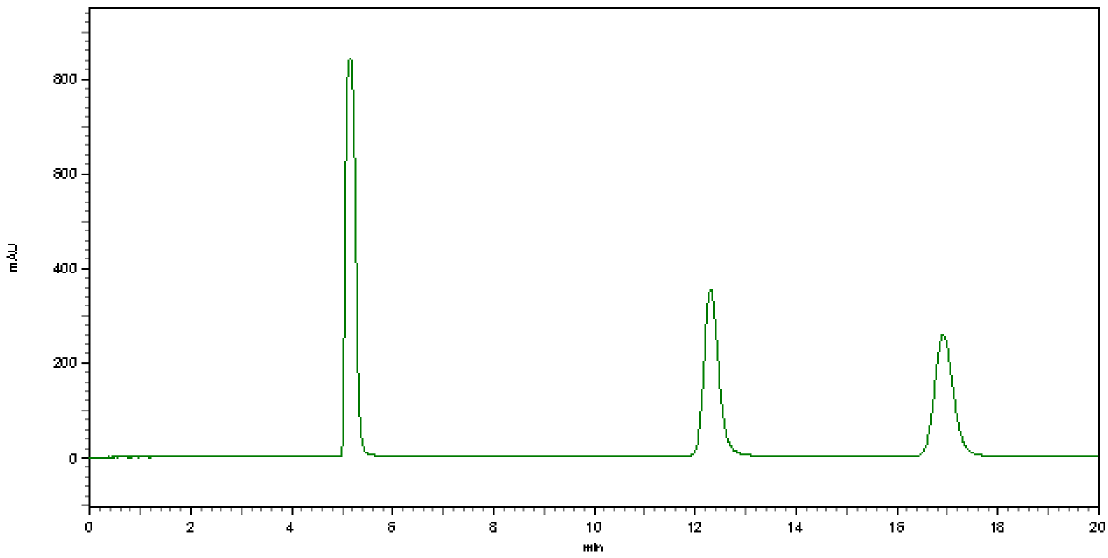

Chiralpak IA column, hexane/2-propanol 90:10, 1.0 mL min^{-1}

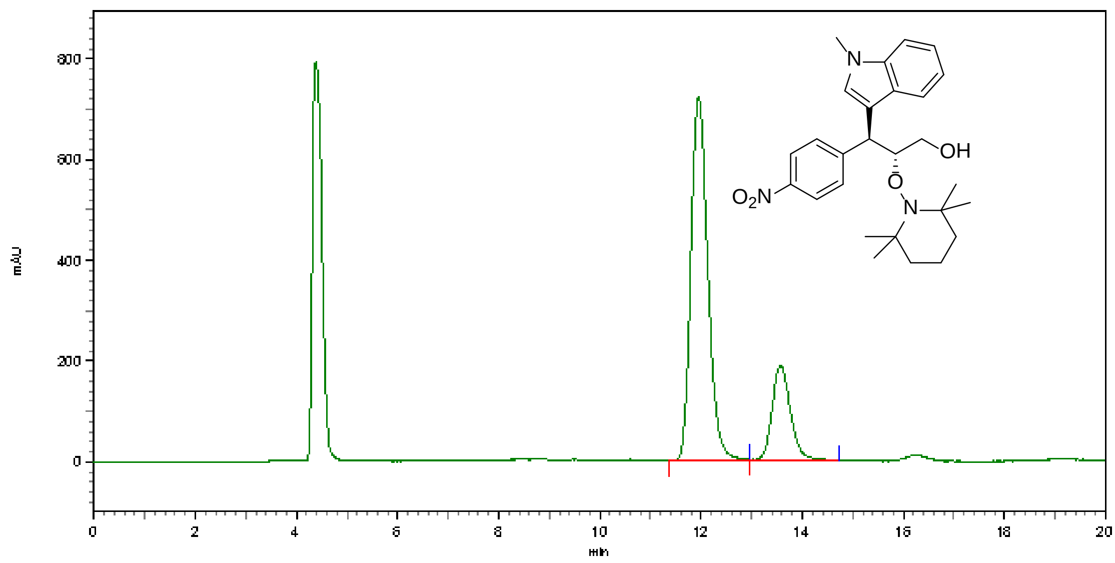

1: 220 nm, 8 nm

retention time	area	area%
11.941	16928427	77.71
13.557	4855259	22.29
Total	21783686	100.00

racemic sample

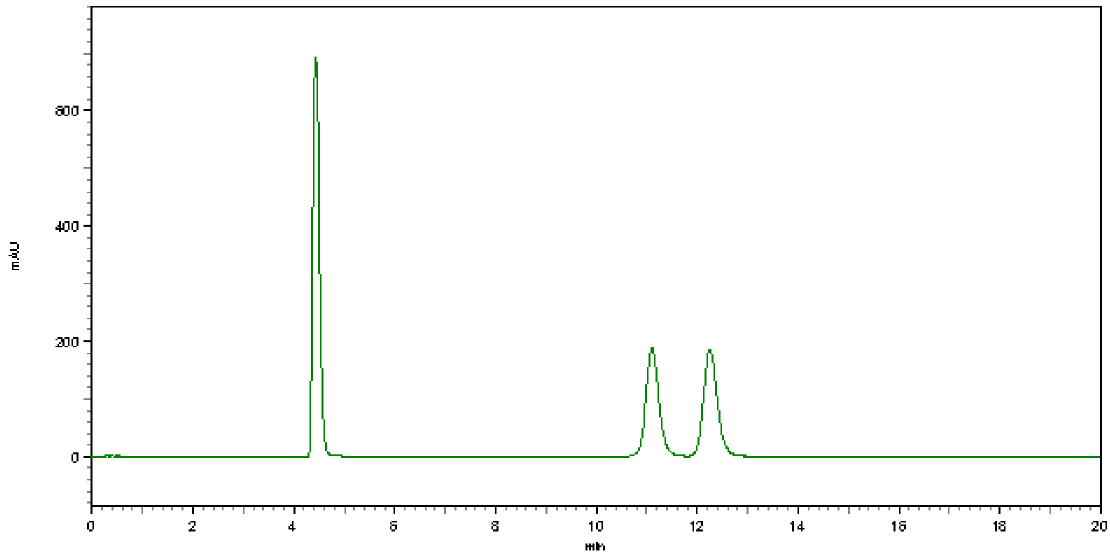

Chiralpak IA column, hexane/2-propanol 90:10, 0.3 mL min^{-1}

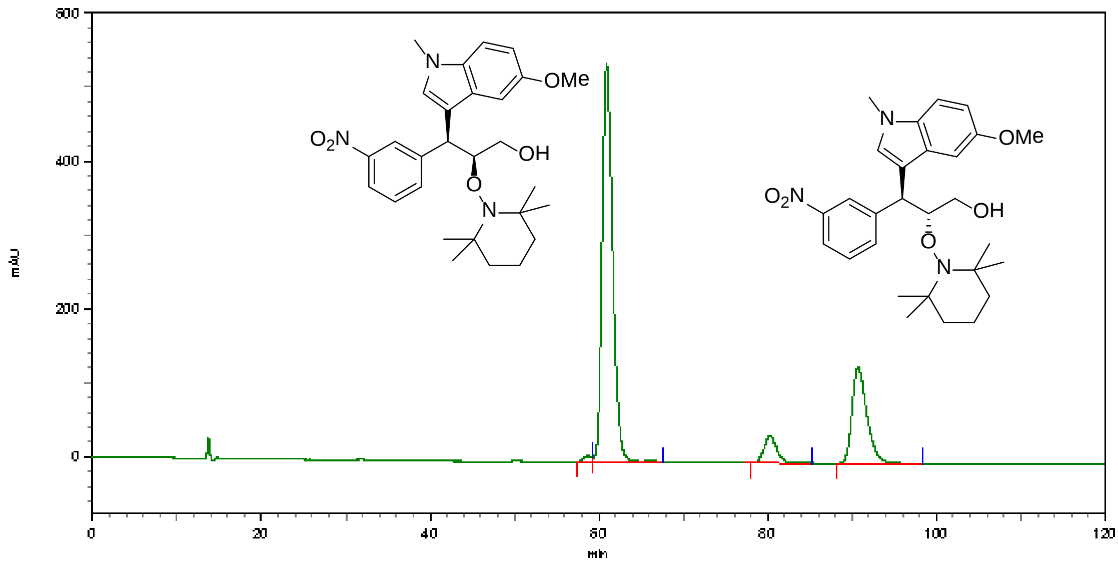

1: 254 nm, 8 nm

retention time	area	area%
58.713	571523	0.85
60.914	46782757	69.59
80.281	3868661	5.76
90.715	15998827	23.80
Total	67221768	100.00

racemic sample

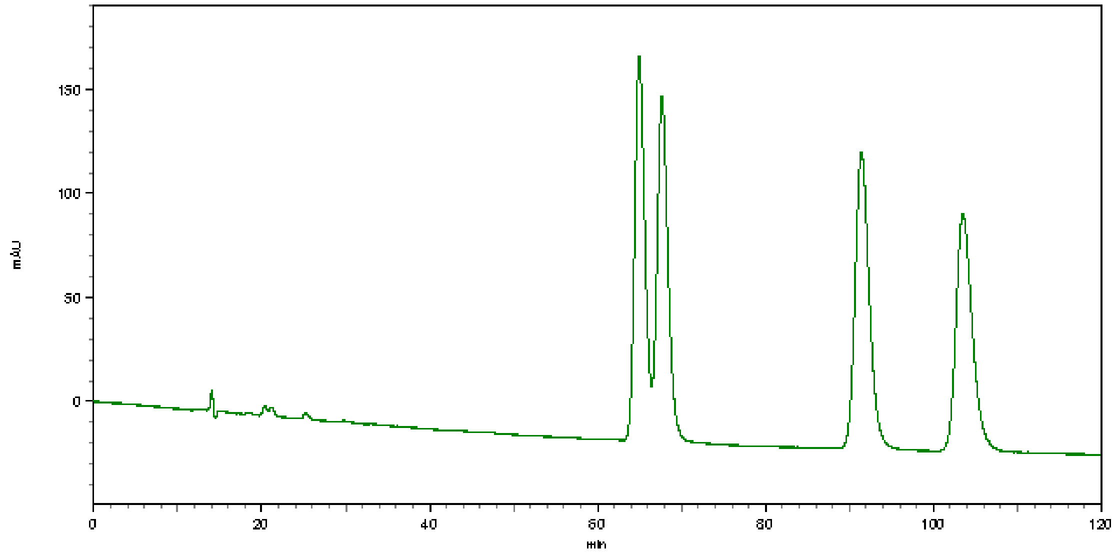

Chiralpak IA column, hexane/2-propanol 90:10, 1.0 mL min^{-1}

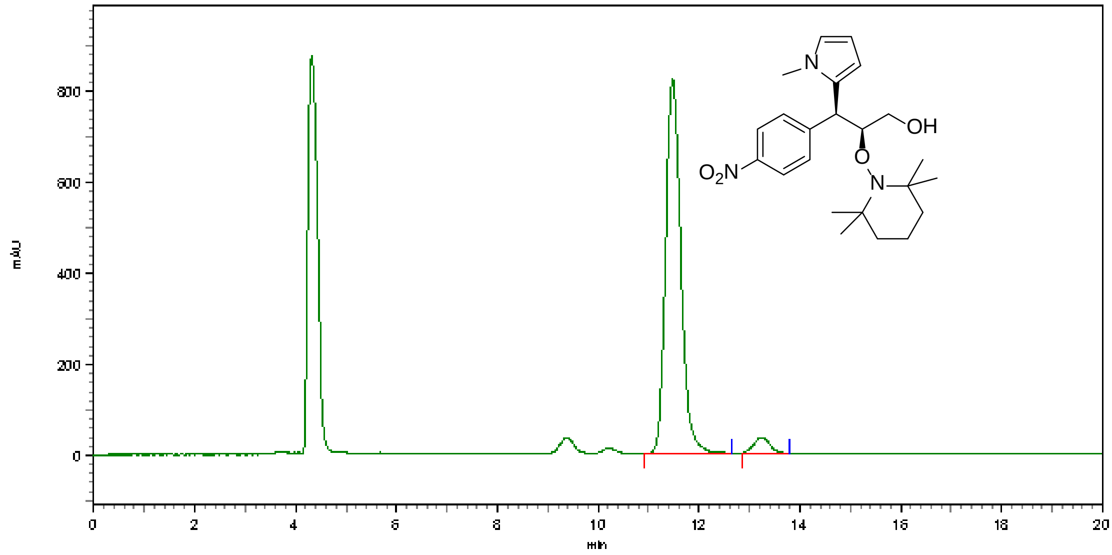

1: 220 nm, 8 nm

retention time	area	area%
11.473	17303582	95.68
13.225	780891	4.32
Total	18084473	100.00

racemic sample

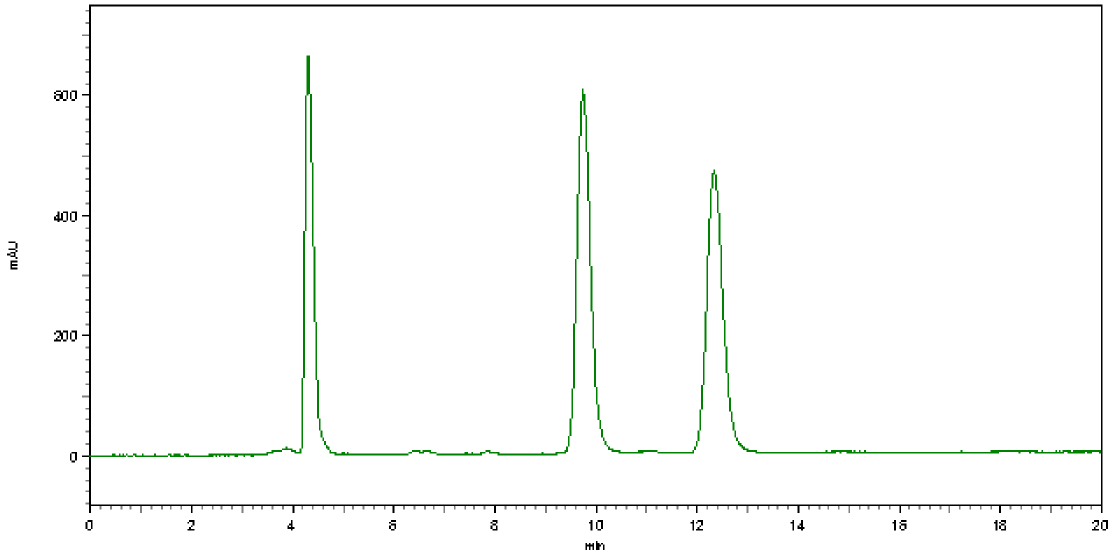

Chiralpak IA column, hexane/2-propanol 90:10, 1.0 mL min^{-1}

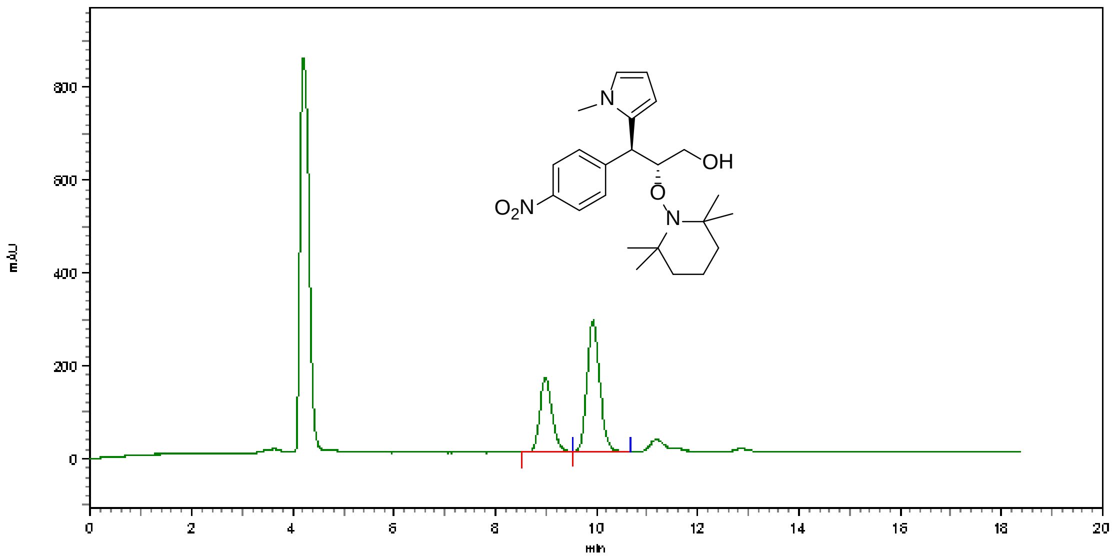

1: 220 nm, 8 nm

retention time	area	area%
8.985	2759167	35.21
9.924	5076678	64.79
Total	7835845	100.00

racemic sample

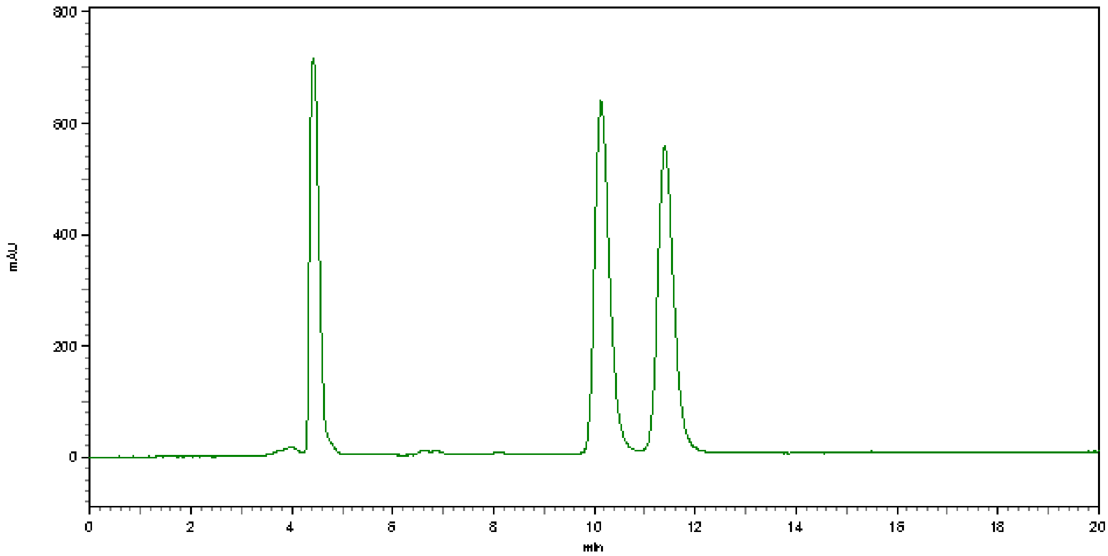

Supplement: File 1 — Typical experimental procedure, spectroscopic data for products, determination of stereochemistry, 1H and 13C NMR spectra and HPLC charts. [file Beilstein_J_Org_Chem-08-1333-s001.pdf]
